# Comprehensive analysis of the mitochondrial genome in lichen genus *Xanthoparmelia*: genetic diversity, intron dynamics, and evolutionary dynamics

**DOI:** 10.3389/fmicb.2025.1740728

**Published:** 2026-01-27

**Authors:** Jinsiguli Bahenuer, Anwar Tumur

**Affiliations:** College of Life Sciences and Technology, Xinjiang University, Urumqi, Xinjiang,China

**Keywords:** intron, mitochondrial genome, phylogeny, repeat sequences, *Xanthoparmelia*

## Abstract

*Xanthoparmelia* (Vain.) Hale is one of the two hyperdiverse genera in the family Parmeliaceae, with over 500 species and a wide distribution. In this study, we sequenced, assembled, and annotated the complete mitochondrial genomes of 11 *Xanthoparmelia* species and conducted a comprehensive analysis to clarify their genetic characteristics and taxonomic status. All 11 mitochondrial genomes consist of circular DNA molecules, with total lengths ranging from 81,194 bp to 88,245 bp and GC contents between 30.2 and 30.8%. Although the *atp9* gene plays a key role in mitochondrial energy production, no atp9 gene was detected in any of the genomes. Additionally, the core genes in the mitochondrial genomes are simplified, which may be a result of coevolution. The results revealed the presence of various dispersed repeats, simple sequence repeats (SSRs), and tandem repeats, which are mainly distributed in intergenic regions and intronic regions. Introns are considered a key factor contributing to variations in mitochondrial genome size. Frequent intron loss/gain events were observed among *Xanthoparmelia* species, further enhancing genome diversity. The Ka/Ks ratios of all 14 protein-coding genes (PCGs) were less than 1, indicating that these genes are under purifying selection and their sequences are evolutionarily conserved. The *cob* gene had the smallest genetic distance, showing high conservation, while the *atp6* gene had the largest genetic distance, indicating a higher mutation rate. Phylogenetic trees of Parmeliaceae were constructed using the maximum likelihood (ML) and Bayesian inference (BI) methods based on the concatenated sequences of 14 PCGs and 2 rRNAs. A well-supported topological structure was generated, clarifying the evolutionary relationships among *Xanthoparmelia* species. This study enriches the mitochondrial genome data of *Xanthoparmelia* and lays a foundation for further understanding the genetic, evolutionary, and phylogenetic relationships of species in this genus.

## 1 Introduction

Lichens are classic symbiotic complexes composed of fungi (symbiotic fungi) and photosynthetic organisms (symbiotic algae or cyanobacteria), and also include associated microorganisms (Zhang et al., 2023), such as bacteria, fungi, and viruses (Grube et al., 2015; Spribille et al., 2016; Li et al., 2025). As the main component of lichen morphological construction, symbiotic fungi play a core role in structural formation, reproductive methods, and secondary metabolism (Nash, 2012). Therefore, the evolutionary process of the fungal genome directly determines the adaptability of lichens and their stable symbiotic relationship with photosynthetic partners, providing an excellent model for studying the origin and evolution of symbiosis. This special symbiotic system enables lichens to have high tolerance in extreme environments, such as waterless deserts or extremely cold regions (Xu et al., 2020), and they play a crucial role in various global ecosystems (Asplund and Wardle, 2017). Lichens exhibit rich morphological and ecological diversity in nature (Brodo et al., 2002). However, evidence based on morphological and chemical characteristics is insufficient to accurately reflect species diversity or define species boundaries. It may even distort the diversity of lichen-forming fungi, especially among closely related species with very similar morphologies. Combining molecular data allows for more accurate species classification (Leavitt et al., 2011; Lumbsch and Leavitt, 2011; Lücking et al., 2021). In recent years, the integration of molecular data has significantly improved the accuracy of species identification and systematic classification. Nevertheless, traditional gene fragment analysis still has limitations. Particularly when inferring geographical origins and reconstructing phylogenetic relationships based on single sequence markers, the results often have high uncertainty. There remain significant uncertainties and controversies regarding the internal taxonomic classification and interspecific phylogenetic relationships of many lichen groups. Although these nuclear genes have been widely used to confirm phylogenetic relationships among species, many species cannot be accurately identified. It is necessary to develop more comprehensive genetic analysis tools to promote in-depth phylogenetic research. With the continuous development of technologies such as high-throughput sequencing, researchers have gradually recognized that phylogenomics is more reliable than single-gene analysis in clarifying species boundaries and evolutionary relationships. Mitochondrial genomes are not only beneficial for solving taxonomic identification problems, especially for symbionts that are difficult to characterize using conventional methods (e.g., arbuscular mycorrhizal fungi) (Nadimi et al., 2016), but also facilitate comparative analysis of mitochondrial genomes to reveal evolutionary events among species, such as dynamic changes in genes (Pogoda et al., 2018).

*Xanthoparmelia* is one of the largest genera in the Parmeliaceae family, with over 800 known species. It is among the two hyperdiverse genera (comprising more than 500 species) (Lücking et al., 2017). This genus exhibits a broad geographical distribution, with two primary centers of distribution located in Australia and southern Africa. Only a small portion of its species are distributed in the Arctic region (Blanco et al., 2004; Crespo et al., 2010; Thell et al., 2012; Leavitt et al., 2018). *Xanthoparmelia* demonstrates considerable morphological and chemical diversity. In recent years, the application of molecular data has expanded the generic boundaries of *Xanthoparmelia*. Meanwhile, it has been indicated that the chemical and morphological characteristics previously used to define intra-generic taxa were overemphasized (Blanco et al., 2004; Leavitt et al., 2011). The widespread cryptic diversity and infraspecific variation within *Xanthoparmelia* have made it more challenging to establish a classification system for this genus based on morphology and chemistry. Research findings have shown that the extensive morphological and chemical similarities within the genus tend to obscure the identification of natural lineages among fungi, whether based on thallus morphology or medullary chemistry (Leavitt et al., 2011). Accurate species delimitation of lichen-forming fungi is crucial for understanding the factors driving diversity and holds significant implications for ecological and conservation studies. As of October 22, 2025, only 4 complete mitochondrial genomes of *Xanthoparmelia* have been published in the NCBI database, and research on the functional analysis of these genomes remains limited. Therefore, there is a distinct gap in mitochondrial-level data in the studies of comparative genomics and phylogenetic resolution of *Xanthoparmelia*.

With the continuous advancement of sequencing technologies, organelle genomes play an important role in species identification, classification, and phylogenetic analysis. Mitochondria are key organelles in fungi and play a vital role in fungal growth, development, and adaptation (Chatre and Ricchetti, 2014). The fungal mitochondrial genome typically consists of a circular DNA molecule and usually comprises 15 protein-coding genes (PCGs). These genes encode 3 ATP synthase subunits, 7 NADH dehydrogenase subunits, 1 complex III (cytochrome c reductase), 3 complex IV (cytochrome c oxidase), and 1 ribosomal protein (*rps3*), which are often present in clusters (Sandor et al., 2018). Mitochondria generate energy through oxidative phosphorylation and are further involved in metabolism, aging, and programmed cell death (Chan, 2006). The fungal mitochondrial genome is characterized by a small size, conserved homologous genes, high copy number, low recombination rate, and high evolutionary rate. These features contribute to its utility in phylogenetic and evolutionary studies (Santamaria et al., 2009; Basse, 2010; Nie et al., 2019). The size of the fungal mitochondrial genome varies significantly, ranging from 12.055 kb (James et al., 2013) to 332 kb (Zaccaron and Stergiopoulos, 2021). Differences in mitochondrial genome size have also been reported among closely related species (Joardar et al., 2012). Such size variations between fungal mitochondrial genomes can usually be explained by the number and size of introns in their genes (Deng et al., 2018). Smaller mitochondrial genomes typically contain no or only a small number of introns, while larger ones are rich in mitochondrial introns (Aguileta et al., 2014; Mardanov et al., 2014; Zubaer et al., 2018). Gene order, repetitive sequences, and in some cases, other types of elements (such as plasmid insertions) are additional sources of variation between fungal mitochondrial genomes (Aguileta et al., 2014). The most notable feature is that although the gene content is largely conserved, the relative gene order is highly variable both between and within major fungal phyla (Paquin et al., 1997). Compared with animals or plants, research on the fungal mitochondrial genome is relatively limited. However, it has great potential in elucidating the evolution of organelle genomes. Clarifying the characteristics of the fungal mitochondrial genome, including the differences in these characteristics among various species, will enable a more comprehensive understanding of the phylogenetic and evolutionary relationships of fungi.

This was investigated through a multi-faceted approach: Firstly, the mitochondrial genome characteristics of the 11 *Xanthoparmelia* species were described. Secondly, a comprehensive comparative analysis was performed to investigate the differences or similarities in genome size, gene arrangement, and repetitive sequences among these mitochondrial genomes. Furthermore, the dynamic changes of introns in the mitochondrial genomes of *Xanthoparmelia* were identified. Finally, phylogenetic analysis was carried out based on 14 PCGs and 2 rRNAs genes to further determine the evolutionary position and topological structure of *Xanthoparmelia* within the phylogenetic tree of Parmeliaceae. This study contributes to a deeper understanding of the genomic evolution, phylogeny, and genetic diversity of mitochondria in the genus *Xanthoparmelia*.

## 2 Materials and methods

### 2.1 Sample collection and sequencing

Eleven species of the genus *Xanthoparmelia* were collected from different locations in Xinjiang, China, external morphologies are shown in Supplementary Figure S1. Detailed information regarding their collection sites and accession numbers is provided in Supplementary Table S1. Species identification was conducted through morphological analysis, internal anatomical examination, chemical characterization, and phylogenetic analysis of the Internal Transcribed Spacer (ITS) sequences in Supplementary Figure S2. (1) Morphological observation: Color, cracks, and luster of the upper surface of the thallus; shape of lobes; presence or absence of white spots; observation of structures such as isidia, pruina, and soralia, as well as characteristics of pycnidia; color of the lower surface; color of rhizines and their branching status (if any). (2) Anatomical observation: Color of the upper and lower cortices of the thallus; uniformity of the algal layer distribution; color of the medulla; observation of structural characteristics of reproductive organs including apothecia and pycnidia.(3) Spot test: Application of chemical reagents to the cortex and medulla of the thallus, followed by observation of color changes. All specimens are preserved in the Cryptogamic Herbarium of the College of Life Science and Technology, Xinjiang University (Contact person: Anwar Tumur, email: awartumursk@xju.edu.cn). DNA extraction was performed using a fungal DNA extraction kit (Sangon Biotech, Shanghai, China) following the manufacturer’s instructions.

### 2.2 Mitochondrial genome assembly and annotation

Whole-genome sequencing of the species was carried out on the DNBSEQ sequencing platform (Shanghai, China). The mitochondrial genomes were *de novo* assembled using GetOrganelle V1.7.4.1 (Jin et al., 2020), a tool specifically optimized for assembling circular organelle genomes. In addition, NOVOPlasty V4.2 (Dierckxsens et al., 2017) was used as an auxiliary assembler. Both tools have been widely and successfully applied in fungal mitochondrial genome studies (e.g., Wu et al., 2021; Anwar et al., 2023). The determination of the circular structure of our mitochondrial assemblies was supported by the following evidence: (1) Published mitochondrial genomes of closely related lichen-forming fungi within the family Parmeliaceae are almost exclusively circular, with no linear mitogenomes reported to date. This provides a strong expectation that *Xanthoparmelia* species share a similar genomic architecture (e.g., Funk et al., 2018; Bahenuer et al., 2025). (2) The assembly logs generated by both GetOrganelle and NOVOPlasty explicitly annotated all 11 assembled mitochondrial contigs as “circular.” (3) We remapped the sequencing reads to the final assemblies and used SAMtools V1.19.2 depth to assess coverage. As shown in Supplementary Figure S3, read depth is uniform and continuous across the entire genome, including the inferred circular-junction regions, without any detectable drops or gaps. These results collectively support both the completeness of our mitochondrial genome assemblies and the reliability of their circular structure. Subsequently, genome annotation was performed on the assembled sequences using the online tools GeSeq V2.03 (Tillich et al., 2017), MFannot V1.3.3 (Valach et al., 2014), and MITOS2 (Bernt et al., 2013) (available at https://usegalaxy.eu). The annotation results were then imported into Geneious V2022.1.1 (Kearse et al., 2012) to check the start and stop codons of protein-coding genes. Finally, the mitochondrial genome map was generated using OGDraw V1.2 (Lohse et al., 2007).

### 2.3 Assembly quality assessment

To evaluate the completeness and uniformity of the newly assembled mitochondrial genomes, whole-genome sequencing depth was calculated with SAMtools V1.19.2 (Li et al., 2009) after read mapping via BWA-MEM V0.7.17 (Li and Durbin, 2009). Coverage plots were generated in R V4.3.1; raw depth is shown in grey and a 201-bp rolling mean (zoo package; Zeileis and Grothendieck, 2005) in red in Supplementary Figure S3. All assemblies exhibited mean depth ≥ 450 × with no region < 10×, confirming genome completeness.

### 2.4 Analysis of repetitive sequences

Repetitive sequences are the main cause of genetic recombination and variation. To evaluate the types and distribution of repetitive sequences in mitochondrial genomes, this study analyzed intragenomic repeats, tandem repeats, interspersed repeats, and simple sequence repeats (SSRs) within the genomes. Local BLASTn (Basic Local Alignment Search Tool, BLASTn) (Chen et al., 2015) was used to align each mitochondrial genome against itself. Tandem repeats in mitochondrial genomes were identified using the online tool Tandem Repeats Finder V4.0 (Benson, 1999) (https://tandem.bu.edu/trf/trf.html). Interspersed repeats in mitochondrial genomes were detected via the online software REPuter (Kurtz, 2001) (https://bibiserv.cebitec.uni-bielefeld.de/reputer) with the following parameters: Hamming Distance = 3, Maximum Computed Repeats = 5,000, and Minimal Repeat Size = 30. MISA (Beier et al., 2017) (https://webblast.ipk-gatersleben.de/misa/) was employed to detect SSRs, with the criteria: 10 repeats for mononucleotides, 5 repeats for dinucleotides, 4 repeats for trinucleotides, and 3 repeats for tetra-, penta-, and hexanucleotides. Finally, TBtools V 2.357 (Chen et al., 2020) was used to visualize the distribution of repetitive sequences.

### 2.5 Analysis of codon usage

PhyloSuite V1.2.2 (Zhang et al., 2020) was used to extract PCGs from each mitochondrial genome. Subsequently, MEGA V11 (Tamura et al., 2021) was applied to calculate the relative synonymous codon usage (RSCU) of the PCGs in the mitochondrial genomes.

### 2.6 Intron analysis

Introns in fungal mitochondria exhibit significant variation. Most eukaryotic mitochondrial genomes typically contain no introns, while species of the genus *Xanthoparmelia* (within Parmeliaceae) usually harbor varying numbers of introns (Martin et al., 2007; Zhang et al., 2015; Chen et al., 2021). Following the reported method (Chen et al., 2021), introns in the PCGs of *Xanthoparmelia* mitochondrial genomes were classified into Pcls (Zhang and Zhang, 2019) using the reference genome of *Tolypocladium inflatum* (NC036382). The specific steps were as follows: PCG without introns were aligned with the reference mitochondrial sequence using MAFFT. Each Pcl consists of introns inserted at the same position in the coding region of a PCG (Cheng et al., 2021). Introns with the same Pcl generally share high sequence similarity and are considered homologous (Férandon et al., 2010). Most different Pcls have low sequence similarity and are associated with non-homologous mobile genetic elements (Huang et al., 2021). The 14 PCG-containing genomes were named based on the insertion positions of introns in the coding regions of the reference genes.

### 2.7 Comparative mitochondrial genomic analysis

Comparative genomic analysis was performed on 11 newly sequenced mitochondrial genome sequences from this study and 4 *Xanthoparmelia* mitochondrial genome sequences downloaded from NCBI in Supplementary Table S2. DNASTAR Lasergene V7.1 (https://www.dnastar.com/software/lasergene/) was used to analyze the base composition of *Xanthoparmelia* mitochondrial genomes. The calculation formulas were: AT skew = [A - T] / [A + T], GC skew = [G - C] / [G + C]. To evaluate the evolutionary rate of coding genes, DnaSP V6.12.03 (Rozas et al., 2017) was used to analyze 14 PCGs, calculating the non-synonymous substitution rate (Ka), synonymous substitution rate (Ks), and their ratio (Ka/Ks). The Ka/Ks ratio indicates the type of selection pressure acting on a gene: A ratio > 1 indicates positive selection. A ratio = 1 indicates neutral evolution. A ratio < 1 indicates purifying selection. MEGA V11 was used to calculate the genetic distances of the 14 PCGs based on the Kimura 2-parameter (K2P) substitution model. Collinearity was analyzed using Mauve (Darling et al., 2004).

### 2.8 Phylogenetic analysis

In this study, maximum likelihood (ML) and Bayesian inference (BI) methods were used to construct a phylogenetic tree of Parmeliaceae based on 14 PCGs. *Lecanora saxigena* (NC_042183) was used as the outgroup. The specific workflow was as follows: Sequences were aligned using MAFFT V7.313 (Katoh et al., 2019). Conserved sequences were filtered using Gblocks V0.91 (Castresana, 2000). The 14 PCGs were concatenated using Sequence Matrix (Vaidya et al., 2011). The most suitable evolutionary model for the dataset was selected using Model Finder (Kalyaanamoorthy et al., 2017). ML analysis was performed using IQ-tree V1.6.8 (Nguyen et al., 2015) with the parameters: -m MFP -bb 1,000 -nt AUTO. Bayesian phylogenetic inference was conducted using MrBayes V3.2.7a (Ronquist et al., 2012) with 2 parallel runs (1,000,000 generations). The initial 25% of run results were discarded (burn-in = 0.25) to construct the BI tree. The phylogenetic trees were visualized and edited using Figtree v1.4.4. (http://tree.bio.ed.ac.uk/software/figtree/).

## 3 Results

### 3.1 Basic characteristics of the mitochondrial genome

**Figure 1:**
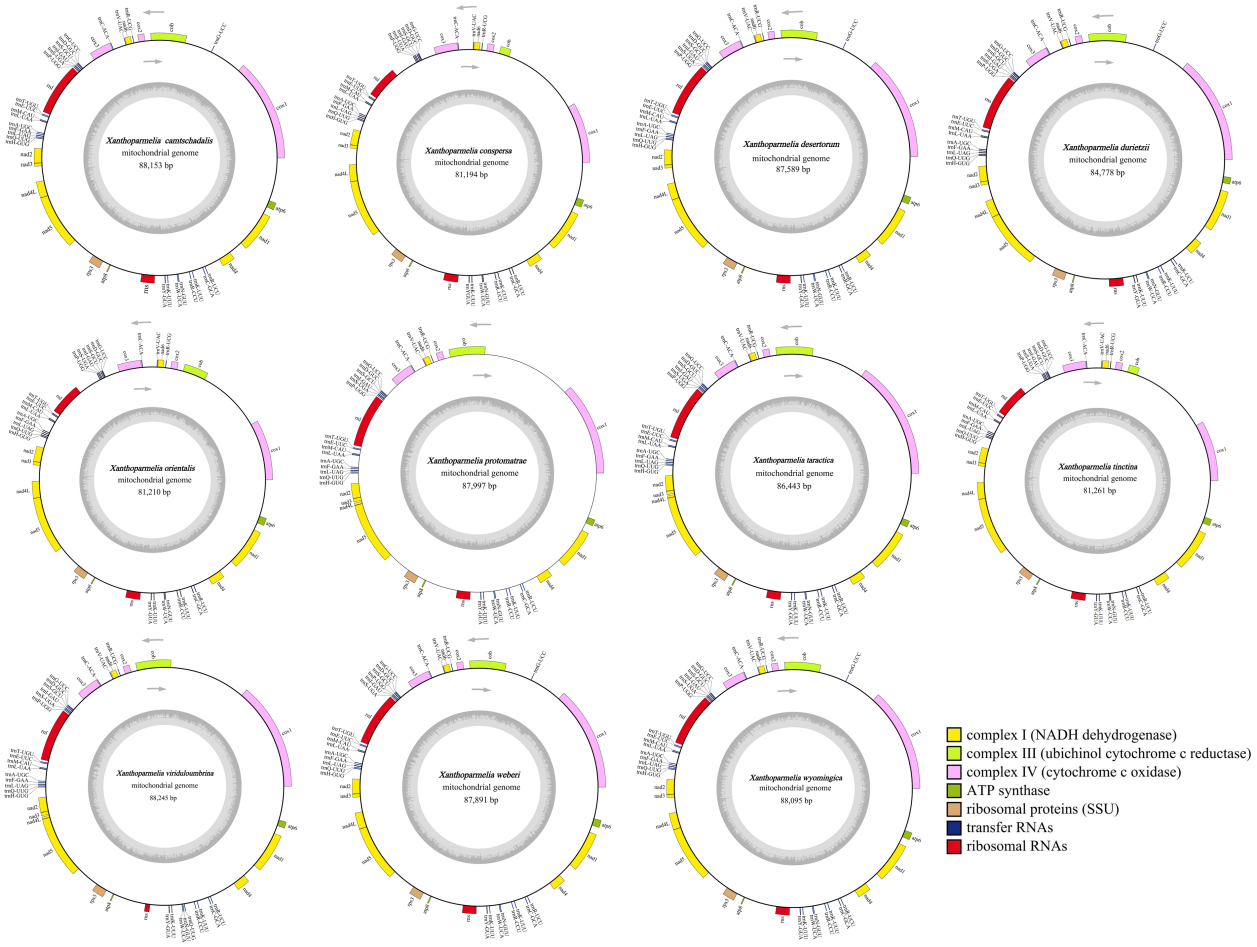
Mitochondrial genome maps of 11 *Xanthoparmelia* species. Different colors represent PSGs with different functions. Genes inside the circle are located on the positive strand, while those outside are on the negative strand. Genes containing introns are marked with an asterisk.

The mitochondrial genomes of all 11 *Xanthoparmelia* species consist of circular DNA molecules, with sizes ranging from 81,194 bp to 88,245 bp (Figure 1). Among them, *X.viriduloumbrina* has the largest mitochondrial genome, while *X.conspersa* has the smallest. The GC content varies from 30.2 to 30.8%, with an average of 30.6%. The AT skews are all negative (−0.006 to −0.013), and the GC skews are all positive (0.057 to 0.063).

Each mitochondrial genome contains 14 PCGs, including 7 NADH dehydrogenase genes (*nad1*, *nad2*, *nad3*, *nad4*, *nad4L*, *nad5*, *nad6*), 3 cytochrome oxidase genes (*cox1*, *cox2*, *cox3*), 2 ATP synthase genes (*atp6*, *atp8*), 1 cytochrome b gene (*cob*), and 1 ribosomal protein subunit 3 gene (*rps3*). Except for *rps3*, these PCGs are conserved mitochondrial genes involved in the oxidative phosphorylation pathway (Li et al., 2020). In addition, two types of ribosomal RNA genes (*rns*, *rnl*) are present in all 11 *Xanthoparnelia* genomes. The number of tRNA genes ranges from 26 to 27 (Supplementary Table S3).

### 3.2 Repeat element analysis

**Figure 2:**
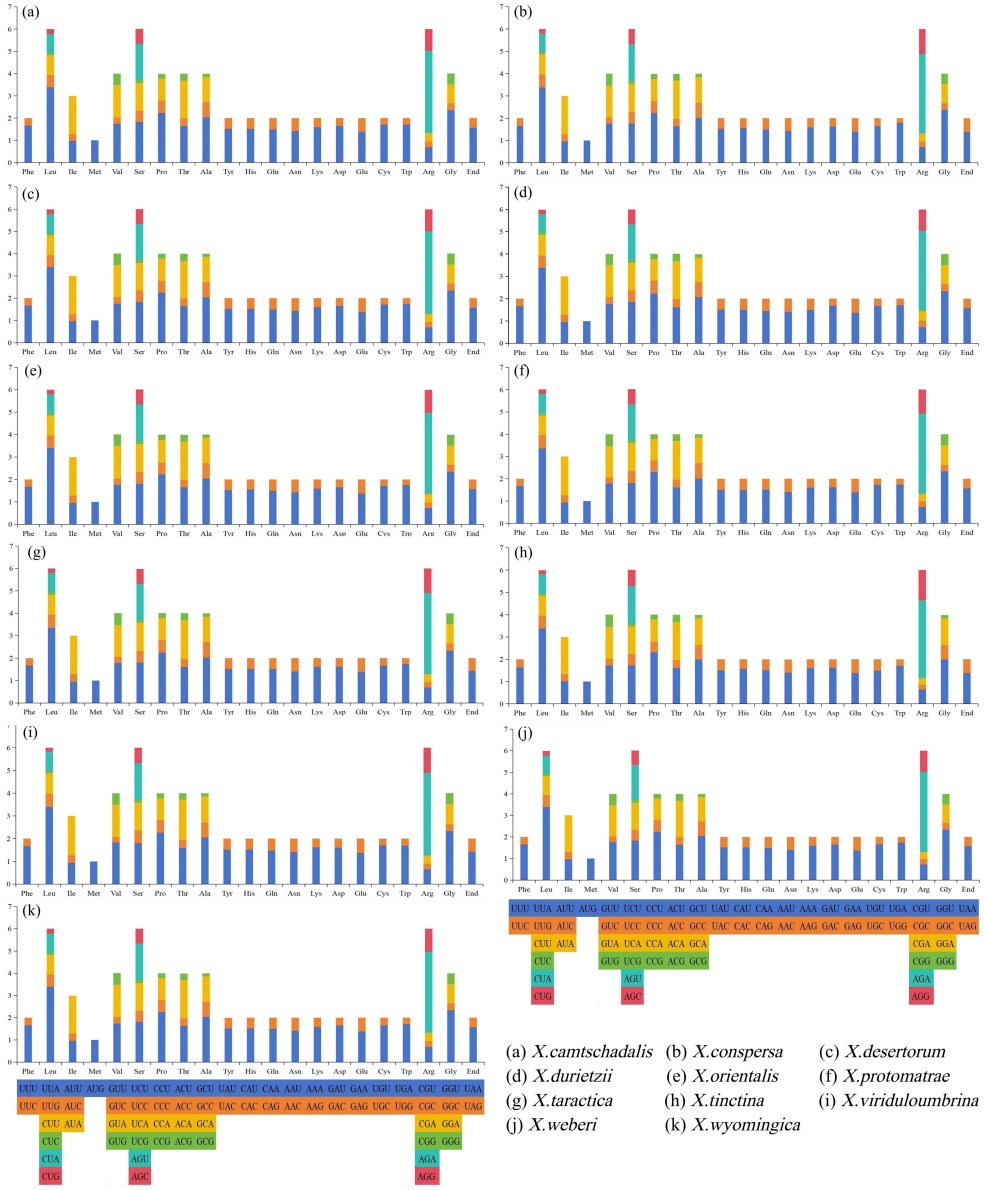
Codon usage analysis of 11 split genomes in the genus *Xanthoparmelia*. The *x*-axis includes 20 standard amino acids encoded by proteins, with the corresponding coding codons labeled below each amino acid. The *y*-axis represents the codon usage frequency.

**Figure 3:**
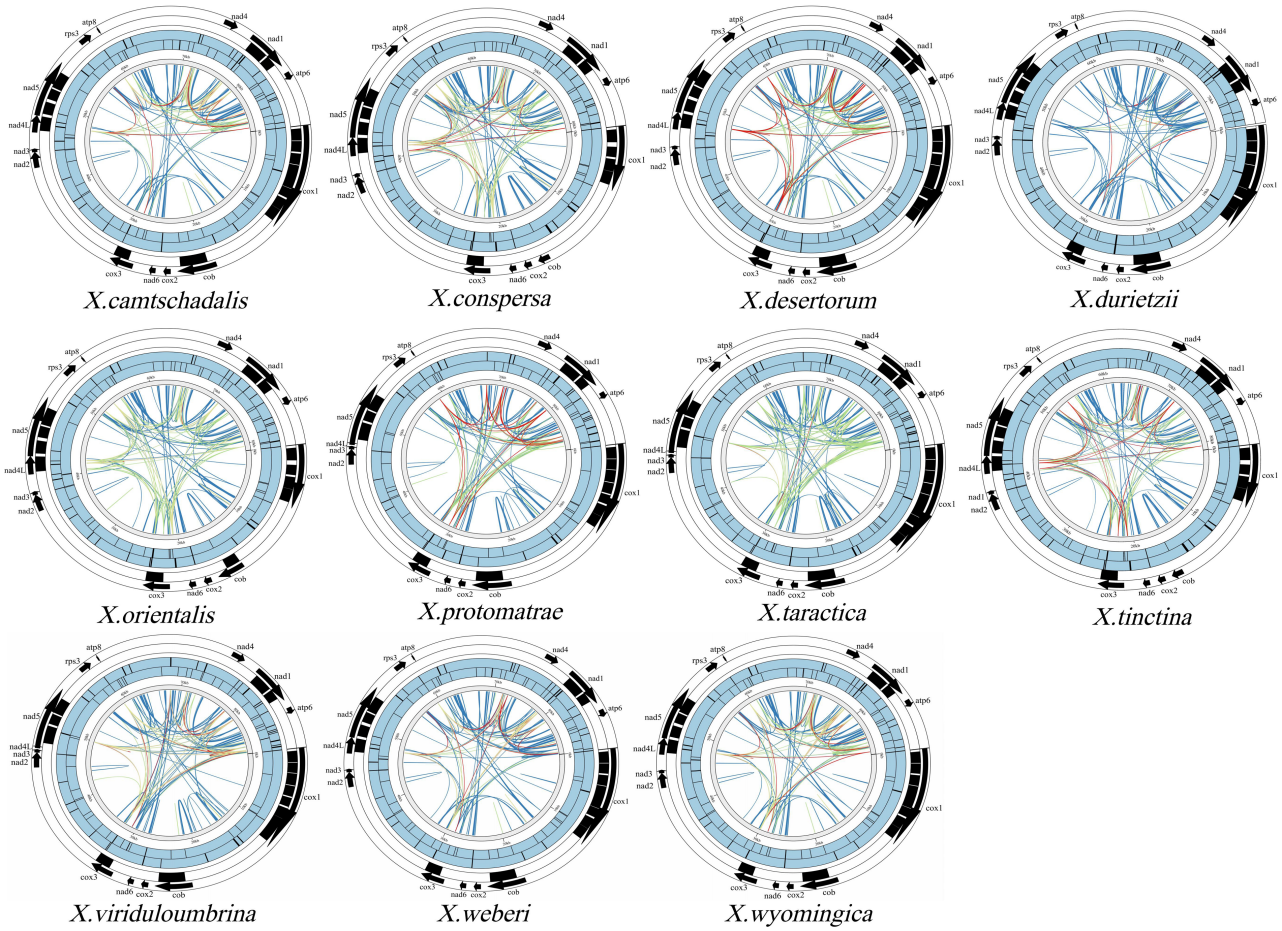
Distribution map of 11 repeat sequences in *Xanthoparmelia* genus. From the inside to the outside, each circle represents: Dispersed repeats (blue lines indicate direct repeats, green lines indicate palindromic repeats, yellow lines indicate inverted repeats, red lines indicate complementary sequences); Simple repeats; Tandem repeats; Introns; Mitochondrial genome coding regions. Black squares represent the covered regions of each element, and blue filling has no specific meaning.

Through BLASTn alignment of the mitochondrial genomes of 11 *Xanthoparmelia* species, we identified 29, 36, 32, 20, 32, 25, 25, 36, 23, 29, and 29 repeat elements in the mitochondrial genomes of *X.camtschadalis, X.conspersa, X.desertorum, X.durietzii, X.orientalis, X.protomatrae, X.taractica, X.tinctina, X.viriduloumbrina, X.weberi, and X.wyomingica*, respectively (Supplementary Table S4). There were 385–457 dispersed duplications (Supplementary Table S5) with repeat sequence sizes ranging from 30 to 281 bp, including 199–256 direct repeats, 71–98 palindromic repeats, 54–96 inverted repeats, and 20–42 complementary repeats. Additionally, there were 51–62 simple sequence repeats (SSRs) (Supplementary Table S6) with sizes ranging from 10 bp to 144 bp. Among these, direct repeats and palindromic repeats exhibited high repetition frequencies across all 11 species. The SSRs included 19–23 mononucleotides, 9–13 dinucleotides, 7–17 trinucleotides, 5–7 tetranucleotides, 2–3 pentanucleotides, and 2–4 hexanucleotides. Furthermore, 48–60 tandem repeats were detected (Supplementary Table S7) with repeat unit sizes of 3–34 bp and copy numbers ranging from 1.9 to 24.2 (Figure 2). SSR markers are commonly used as molecular markers in genetic diversity and evolutionary studies (Ahmad et al., 2018). Analysis of the distribution of repeat sequences across the 11 mitochondrial genomes revealed that they are mainly located in introns and intergenic regions (Figure 3).

### 3.3 Codon usage analysis

The start codons and stop codons of 14 PCGs in the mitochondrial genomes of 11 *Xanthoparmelia* species were compared (Supplementary Table S8). Among the 14 PCGs: The *atp8*, *cox1*, *cox3*, *nad1*, *nad2*, *nad3*, *nad4L*, and *nad5* genes used ATG as their start codon. Both *cox2* and *rps3* genes used TTA as the start codon. The *atp6* gene used ATG and ATA as start codons. *nad4* and *nad6* used ATC and ATG as start codons, respectively.

Most species used ATG as the start codon for the *cob* gene, except *X.wyomingica* and *X.viriduloumbrina*, which used ATC and ATA, respectively. For stop codons: The *atp6*, *cob*, *cox2*, *cox3*, *nad2*, *nad3*, *nad4*, *nad4L*, *nad6*, and *rps3* genes terminated with TAA. The *atp8*, *cox1*, and *nad1* genes used TAG as the stop codon. Most species used TAA as the stop codon for the *nad5* gene, except *X.taractica* and *X.viriduloumbrina*, whose *nad5* genes terminated with TAG. Studies have shown that codon usage directly affects translation speed and energy requirements. Genes in mitochondria are thought to prefer specific codons to save time and conserve energy needed for cell growth (Sharp, 2005). The codon usage analysis results revealed that the PCGs in the mitochondrial genomes of 11 *Xanthoparmelia* species exhibited highly similar codon usage preferences (Figure 2). Among the analyzed amino acids: Leucine (Leu), serine (Ser), and arginine (Arg) were each encoded by 6 codons. Methionine (Met) was encoded by only 1 codon. The codons UUU (for Phe), UUA (for Leu), AUA (for Ile), and UAU (for Tyr) had the highest usage frequency across the 11 mitochondrial genomes (Supplementary Table S9). The mitochondrial genomes of *Xanthoparmelia* showed a high AT content, mainly due to the high-frequency use of A/T bases in their preferred codons. Arginine (Arg) in *Xanthoparmelia* mitochondrial genomes was primarily encoded by the AGA codon. The results indicated that, except for AGA, the Relative Synonymous Codon Usage (RSCU) values of all other arginine codons (CGU, CGC, CGA, AGG) were below 1, with CGG having an RSCU value of 0. Meanwhile, the RSCU value of UUA (for Leu) was above 3, which may be associated with environmental stress.

### 3.4 Analysis of intron dynamics repeat element analysis

**Figure 4:**
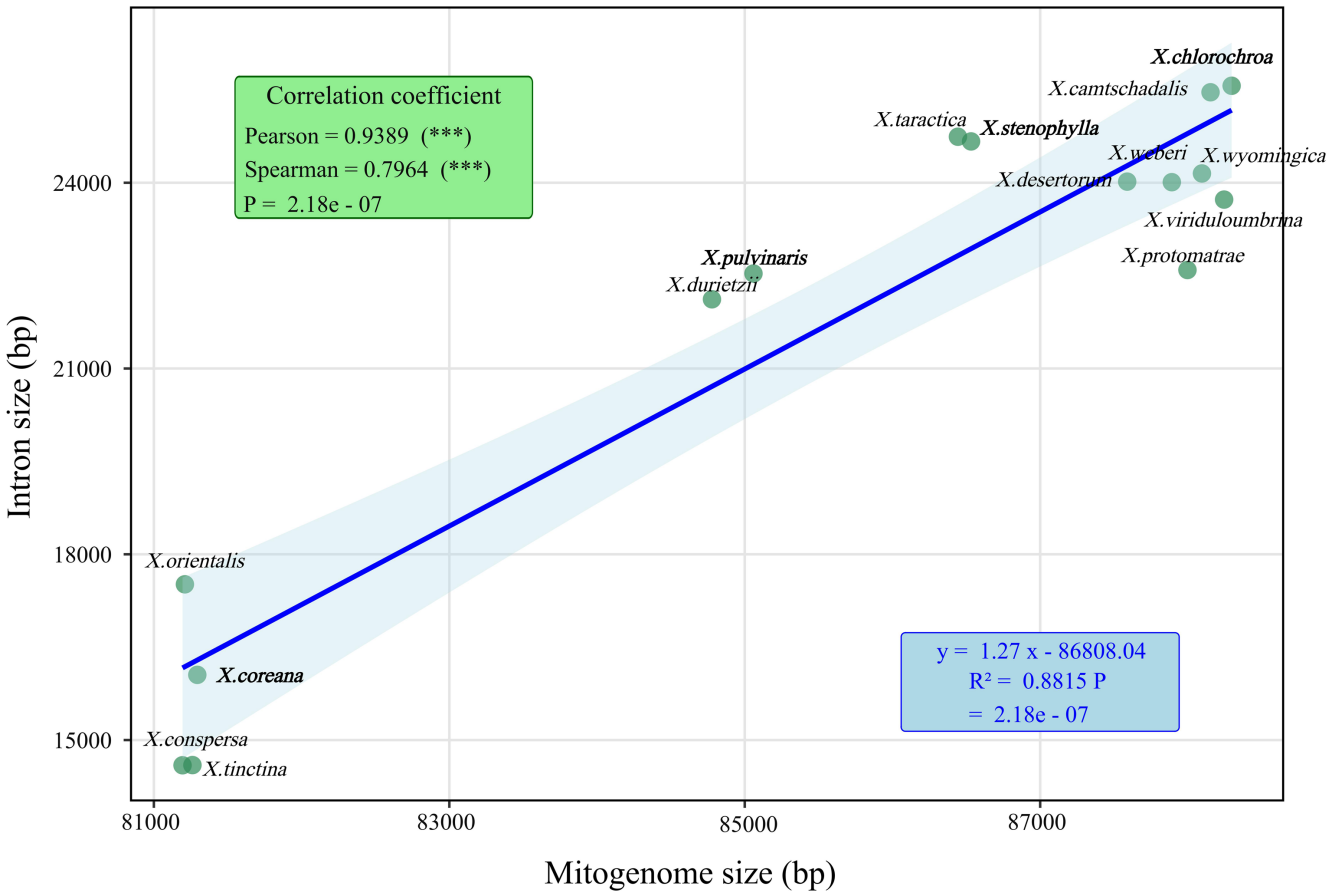
Correlation analysis between intron length and mitochondrial genome size of 15 *Xanthoparmelia* species. The asterisks in the figure indicate statistical significance: *p* < 0.05 (significant), *p* < 0.01 (highly significant), *p* < 0.001 (extremely significant). The 4 species downloaded from NCBI are shown in bold.

There was a significant correlation between intron size and the size of 15 *Xanthoparmelia* mitochondrial genomes (*p* < 0.001). The Pearson and Spearman correlation coefficients were 0.9389 and 0.7964, respectively, indicating that intron length has a significant impact on the variation in the size of *Xanthoparmelia* mitochondrial genomes (Figure 4). A total of 239 introns were detected across the 15 mitochondrial genomes, with each species containing 11 to 24 introns. Studies have shown that intron loss or gain events occurred throughout the entire evolutionary history of the *Xanthoparmelia* genus. Among these introns, 95% (228 introns) were located in PCGs, and 5% (11 introns) were located in rRNA genes; thus, PCGs are the main reservoirs of introns. Introns were distributed in the *cox1*, *cox3*, *cob*, *nad1*, *nad4L*, and *nad5* genes. This uneven distribution of introns indicates gene preference, with most introns targeting PCGs.

**Figure 5:**
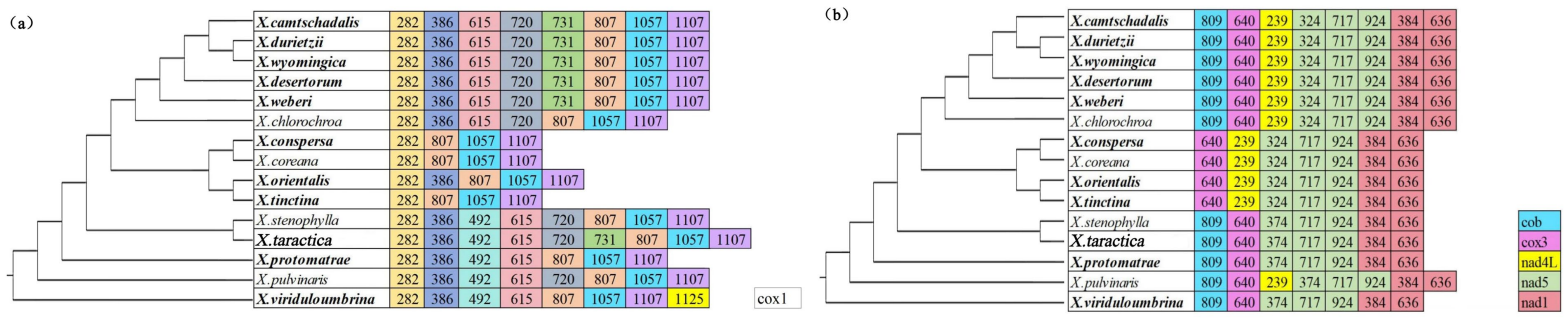
Shows the position classification (Pcl) of introns in the *cox1* gene **(a)** and other PCGs **(b)** across 15 species of the *Anthoparmelia* genus. Pcls (homologous introns) are labeled based on their insertion sites (nucleotide positions) in the reference gene (GenBank Accession Number: NC036382). The phylogenetic positions of the 15 *Xanthoparmelia* species were determined using the Bayesian inference (BI) and Maximum likelihood (ML) methods. The 4 species downloaded from NCBI are shown in bold.

Introns were classified into different positional classes (Pcls) corresponding to their reference genes based on their insertion sites within the protein-coding regions of the mitochondrial genome. Introns in the *cox1* gene of 15 *Xanthoparmelia* species were categorized into P282, P386, P492, P615, P720, P731, P807, P1057, P1107, and P1125, among which P282, P807, P1057, and P1107 had the widest distribution. The *cob*, *cox3*, *nad4L*, *nad5*, and *nad1* genes contained 1, 1, 1, 3, and 2 types of Pcls, respectively. The Pcls of *cox3*, *nad5*, and *nad1* were widely distributed across the 15 *Xanthoparmelia* species. Except for the *cox1* gene, *X.taractica* contained the most intron types, while *X.conspersa*, *X.coreana*, *X.orientalis*, and *X.tinctina* showed significant intron loss in their *cox1* genes, retaining only 4 to 5 intron types (Figure 5). These findings indicate that among the 14 PCGs, the *cox1* gene in the mitochondrial genome of *Xanthoparmelia* species exhibits relatively high variation, and there is a possibility of intron transfer.

### 3.5 Genetic distance and evolutionary rate of PCGs

**Figure 6:**
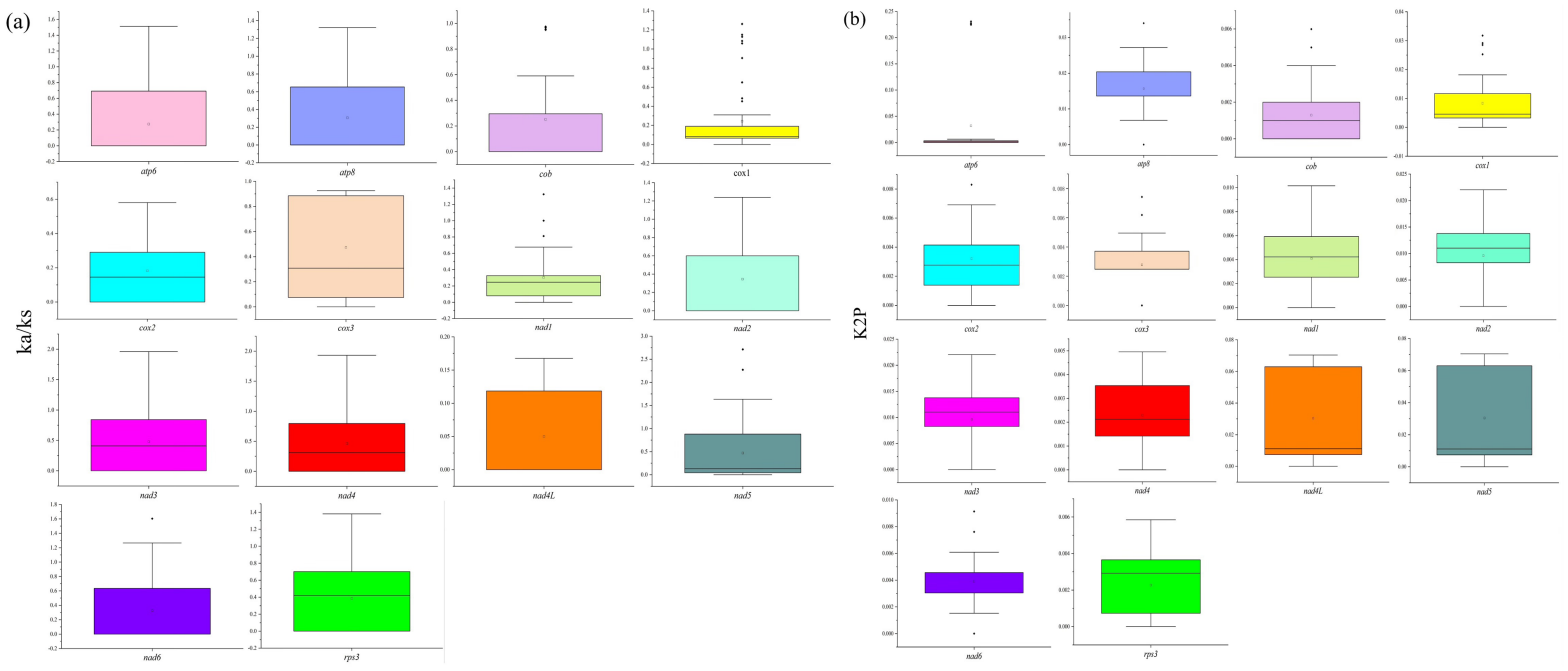
Evolutionary selection pressure **(a)** and genetic distance **(b)** of 14 PCGs in 15 species of the genus *Xanthoparmelia*.

Among the 14 PCGs in the mitochondrial genome of genus *Xanthoparmelia*, the Ka/Ks values of all 14 PCGs are less than 1 (Figure 6a), indicating that these genes are under purifying selection and their sequences evolve more conservatively. The results of genetic distance (K2P) analysis show (Figure 6b) that the 14 PCGs have different genetic distances, which means their evolutionary rates also vary. Among them, the *atp6* gene has the largest genetic distance (with an average value of 0.231), suggesting that it exhibits the fastest mutation rate among the 14 PCGs. In contrast, the *cob* gene has the smallest genetic distance (with an average value of 0.001), indicating that it has high conservativeness.

### 3.6 Synteny analysis

**Figure 7:**
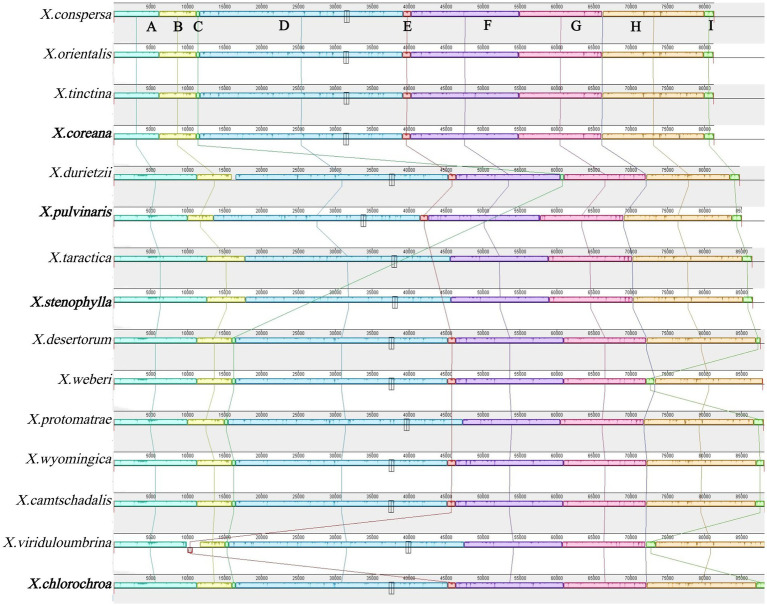
Collinearity analysis of 15 mitochondrial genomes in the genus *Xanthoparmelia*. The analysis identifies homologous regions **(A–I)**, each represented by a unique color block. The four species downloaded from NCBI are shown in bold.

A detailed comparison of collinearity among the 15 mitochondrial genomes revealed a conserved core structure punctuated by specific rearrangements. The analysis identified nine homologous blocks, with the order and orientation of most blocks being stable across the genus (Figure 7). However, detailed comparison revealed several specific rearrangements: (1) The third block (containing *cox1* and *cob*) was rearranged exclusively in *X.durietzii*; (2) The fifth block (intergenic between *nad3* and *nad4L*) was rearranged in *X.viriduloumbrina*; (3) The ninth block underwent independent rearrangements in both *X.weberi* and X.*viriduloumbrina*. This pattern indicates that the sequences of PCGs are highly conserved, while structural variation is confined primarily to intergenic spacer regions, which may facilitate genome evolution without disrupting essential coding functions.

### 3.7 Phylogenetic analysis

**Figure 8:**
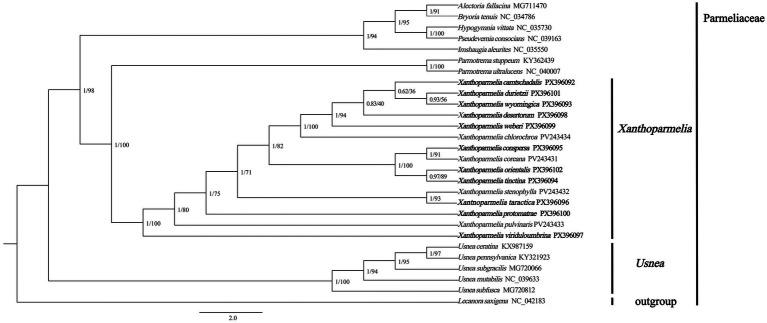
Phylogenetic tree of 27 species in Parmeliaceae. Phylogenetic trees were constructed using 14 PCGs and 2 rRNAs via Bayesian inference (BI) and maximum likelihood (ML) methods, with *Lecanora saxigena* (NC_042183) as the outgroup. The numbers above the nodes represent Bayesian posterior probabilities (left) and bootstrap values (right). Detailed species names and accession numbers of the mitochondrial genomes used in the phylogenetic analysis are available (Supplementary Table S2).

The entire phylogenetic tree was divided into 4 clades, including the genera *Alectoria*, *Bryoria*, *Hypogymnia*, *Imshaugia*, *Parmotrema*, *Pseudevernia* and *Usnea*. The phylogenetic results were consistent with the topological structure reported in previous studies (Funk et al., 2018; Bahenuer et al., 2025). Fifteen species of the genus *Xanthoparmelia* formed a monophyletic clade, which clustered with Parmotrema with high bootstrap support (100%) and posterior probability (1.00). This indicates a close phylogenetic relationship between the two genera, or they may share a common ancestor *X.conspersa*, *X.coreana*, *X.orientalis*, and *X.tinctina* clustered closely into a subclade, while *X.viriduloumbrina* branched closer to the terminal node (Figure 8).

## 4 Discussion

In this study, we assembled and annotated the mitochondrial genomes of 11 species in the genus *Xanthoparmelia* and conducted a comparative analysis of 15 *Xanthoparmelia* species. This analysis revealed the conservation and variability of the genus, providing high-quality data for future research. The mitochondrial genome structure of *Xanthoparmelia* is similar to that of other reported Parmeliaceae species. Notably, although *atp9* plays a key role in mitochondrial energy production, we found that the mitochondrial genomes of *Xanthoparmelia* contain 14 PCGs and lack the *atp9* gene, which differs from most fungal mitochondrial genomes. This may be because symbiosis between coevolving taxa is typically characterized by genome reduction to reduce redundancy or inter-genomic conflicts (Pogoda et al., 2018). The loss of *atp9*—a key energy-related gene—in lichen symbiotic fungi provides genomic evidence for the specificity of symbiotic relationships. This study offers new resources for further investigating the evolution of lichen mitochondrial genomes and their coevolution with specific symbiotic partners.

Previous studies have shown that fungal mitochondrial genome size is one of the most variable among eukaryotes, primarily due to dynamic changes in introns, accumulation of repetitive sequences, and other factors (Basse, 2010; Al-Reedy et al., 2012; Deng et al., 2018; Wu et al., 2021). In this study, we found that introns are unevenly distributed in PCGs and rRNA genes of *Xanthoparmelia*, with the *cox1* gene being the largest host gene for introns. Introns can be classified into different pcls based on their insertion positions, and introns with the same pcl are considered homologous (Li et al., 2020). Intron size has a significant impact on the size variation of *Xanthoparmelia* mitochondrial genomes (*p* < 0.001), indicating that introns may be a key factor driving differences in mitochondrial genome size within this genus. Among the analyzed species, *X.chlorochroa* has the largest mitochondrial genome (88,296 bp) and the longest total intron length (25,566 bp), while *X.conspersa* has the smallest mitochondrial genome (81,194 bp) and the shortest total intron length (14,593 bp). This correlation suggests that intron loss events may have occurred during the evolution of *Xanthoparmelia* mitochondrial genomes, leading to differences in genome size among different species. The physiological and functional effects of these intron dynamics require further investigation.

Our results revealed complete and conserved PCGs across all species. The codon usage frequency and variations in start/stop codons of these PCGs were minimal, indicating that PCGs remain relatively conserved throughout evolution. The Ka/Ks ratios of all 14 PCGs were less than 1, suggesting that all genes have undergone conservative purifying selection during evolution. Synteny analysis showed dynamic changes in the mitochondrial genomes of *Xanthoparmelia* species, manifested as rearrangements between homologous gene clusters, and this phenomenon mainly occurs in intergenic regions. Previous studies have shown that the accumulation of repetitive sequences in fungal mitochondrial genomes is closely related to mitochondrial gene rearrangements (Aguileta et al., 2014).

In fungal evolution research, mitochondrial genes are often important markers for molecular systematics and population genetics (Nadimi et al., 2016). Phylogenetic analysis based on fungal mitochondrial gene fragments has been widely used in lichen taxonomy research and has shown good results in resolving difficult issues such as clarifying species boundaries (Börstler et al., 2008). Therefore, it is widely applied in lichen taxonomy research and analysis (Schmitt and Lumbsch, 2004; Blanco et al., 2005; Crespo et al., 2010; Kondratyuk et al., 2016; Černajová and Škaloud, 2019; Chiva et al., 2021). Compared with single-gene analysis, the use of complete mitochondrial genomes or multi-gene combined analysis can more comprehensively reflect the evolutionary history of genomes (Seifert et al., 2007). Additionally, based on the currently published mitochondrial genome annotation results, lichens still retain most PCGs, which provides favorable conditions for conducting more accurate genetic evolution research. *Xanthoparmelia* is one of the widely distributed and species-rich genera in Parmeliaceae. However, understanding its internal evolutionary relationships has been challenging due to limited morphological characteristics and insufficient multi-locus phylogenetic studies. Therefore, more powerful tools are needed to provide richer genetic information for accurate phylogenetic analysis (Jiang et al., 2017). Through comprehensive comparative analysis of mitochondrial genomes, our phylogenetic study strongly supports the close evolutionary relationships among these 15 *Xanthoparmelia* species. We constructed phylogenetic trees of 27 species using Maximum Likelihood (ML) and Bayesian Inference (BI) methods, and the topological structure of the results received high support, with bootstrap support values exceeding 70% for most branches—a threshold generally considered to represent strong support in phylogenetic research. These results clarify the phylogenetic relationships among species and determine the phylogenetic positions of 11 *Xanthoparmelia* species. Thus, this study provides a more comprehensive and robust phylogenetic framework for the genus, highlighting the importance of mitochondrial genome data in resolving taxonomic uncertainties and clarifying evolutionary relationships within this complex genus. It also provides valuable reference data for species classification and identification, helping to deepen understanding of interspecific variation within the genus and offering new genetic insights into the genetics, systematics, genomics, and evolution of *Xanthoparmelia*.

## 5 Conclusion

This study presents the first comprehensive report of 11 complete mitochondrial genomes within the genus *Xanthoparmelia*, substantially enriching the organelle genomic resources for this hyperdiverse lichen group. Our analysis reveals that the mitochondrial genome size in *Xanthoparmelia* (81–88 kb) is primarily governed by a dual mechanism: a strong positive correlation between intron length and genome size (Pearson = 0.94, Spearman = 0.80, *p* < 0.001) coupled with the variable presence of dispersed repeats, SSRs, and tandem repeats. We identified the *cox1* gene as a dynamic hotspot of genomic diversity, harboring ten Pcl-type introns and exhibiting frequent loss/gain events. All 14 protein-coding genes are under strong purifying selection (Ka/Ks < 1), underscoring the evolutionary conservation of core mitochondrial functions. Notably, the *atp6* gene exhibits the highest interspecific genetic distance (K2P = 0.231) and may serve as a high-resolution molecular marker for future species delimitation. Phylogenomic reconstruction using 14 PCGs and 2 rRNAs yielded a fully resolved and strongly supported topology that clearly delineates the major clades within *Xanthoparmelia*. Taken together, these findings not only close a critical gap in mitochondrial genome data for *Xanthoparmelia* but also provide a foundational resource for: (i) clarifying species boundaries obscured by morphological and chemical similarities, especially when integrated with ITS data; (ii) testing the hypothesis of symbiosis-driven mitochondrial genome reduction; and (iii) supporting future studies in conservation genomics, phylogenetics, and the functional evolution of lichen-forming fungi within Parmeliaceae.

## Data Availability

The datasets presented in this study can be found in online repositories. The names of the repository/repositories and accession number(s) can be found in the article/Supplementary material.

## Supplementary material

SUPPLEMENTARY FIGURE S1 External morphology of eleven *Xanthoparmelia* species.

SUPPLEMENTARY FIGURE S2 Phylogenetic tree of eleven *Xanthoparmelia* species constructed based on ITS sequences.

SUPPLEMENTARY FIGURE S3 Mitochondrial genome sequencing coverage of 11 *Xanthoparmelia* species.
