# Correction: Comprehensive analysis of the mitochondrial genome in lichen genus *Xanthoparmelia*: genetic diversity, intron dynamics, and evolutionary dynamics

**DOI:** 10.3389/fmicb.2026.1803503

**Published:** 2026-02-16

**Authors:** Jinsiguli Bahenuer, Anwar Tumur

**Affiliations:** College of Life Sciences and Technology, Xinjiang University, Urumqi, Xinjiang, China

**Keywords:** intron, mitochondrial genome, phylogeny, repeat sequences, *Xanthoparmelia*

File [Supplementary file 1:Table S1] was erroneously published with the original version of this paper. The file has now been replaced.

**SUPPLEMENTARY TABLE S1:** Specimen collection information.

**Species name**	**Longitude**	**Latitude**	**Elevation**	**GenBank**	**Species number**
*X. camtschadalis*	82°41'28”	43°31'32”	2,219 m	PX396092	JS202408002
*X. conspersa*	83°0'57”	45°49'41”	1,684 m	PX396095	JS202406126
*X. desertorum*	82°54'9”	43°13'4”	1,340 m	PX396098	JS202407002
*X. durietzii*	82°30'43”	45°44'28”	1,313 m	PX396101	JS202406137
*X. orientalis*	83°2'32”	46°4'33”	1,074 m	PX396102	JS202406145
*X. protomatrae*	82°44'21”	45°55'15”	1,179 m	PX396100	JS202406127
*X. taractica*	88°24'10”	43°42'46”	3,049 m	PX396096	JS202407032
*X. tinctina*	82°54'6”	43°13'2”	1,342 m	PX396094	JS202407001
*X. viriduloumbrina*	83°2'54”	46°0'3”	1,085 m	PX396097	JS202406203
*X. weberi*	92°56'4”	43°32'54”	2,121 m	PX396099	JS202407003
*X. wyomingica*	89°36'56”	43°38'16”	1,705 m	PX396093	JS202408001

The original version of this article has been updated.

