# Supplementary figures and images for: Comprehensive analysis of the mitochondrial genome in lichen genus Xanthoparmelia: genetic diversity, intron dynamics, and evolutionary dynamics
Source: Front Microbiol. 2026 Jan 27;16:1740728. doi: 10.3389/fmicb.2025.1740728 (PMC12886410; doi:10.3389/fmicb.2025.1740728)

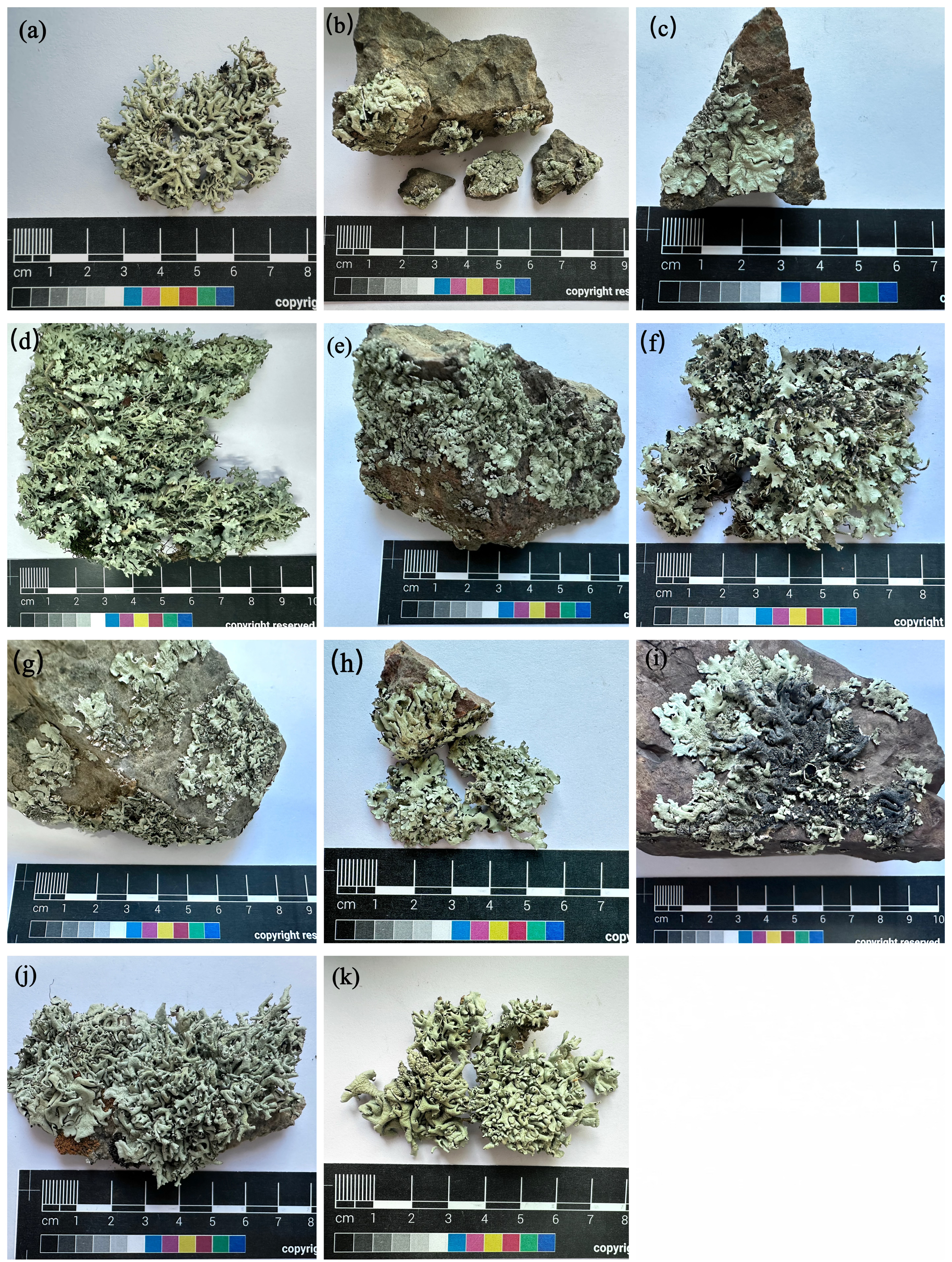

Supplement: SUPPLEMENTARY FIGURE S1 — External morphology of eleven Xanthoparmelia species. [file Data_Sheet_1.zip › Supplementary Figures/Supplementary Figure 1/Figure 1.jpg]

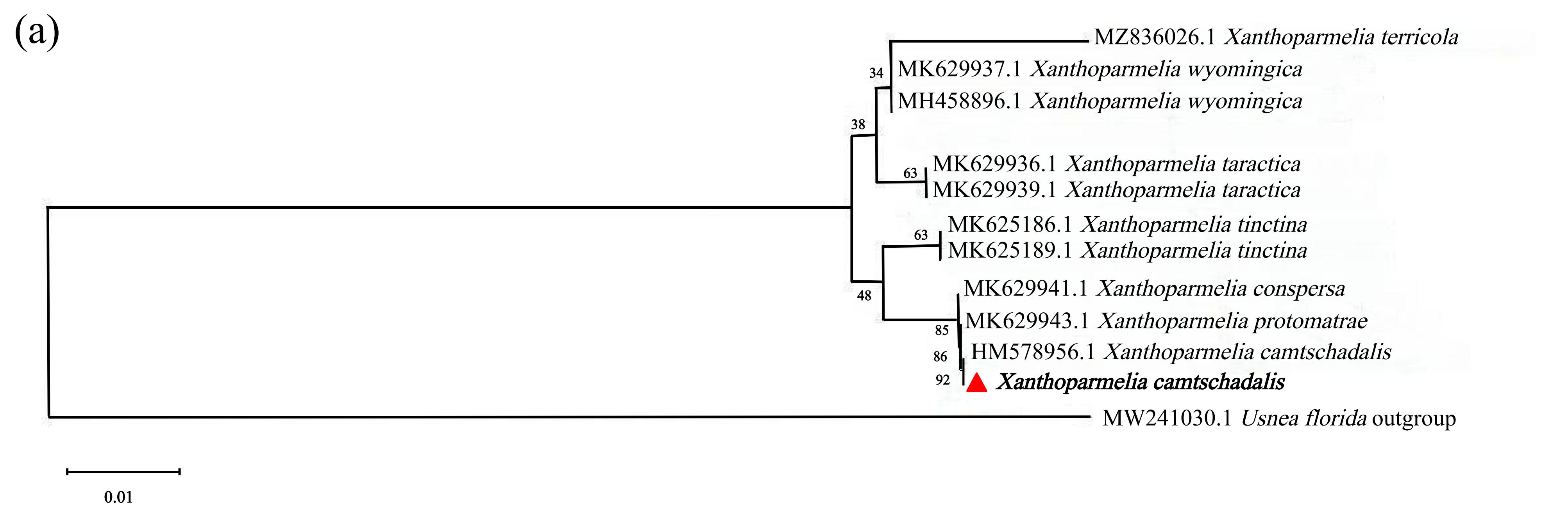

Supplement: SUPPLEMENTARY FIGURE S1 — External morphology of eleven Xanthoparmelia species. [file Data_Sheet_1.zip › Supplementary Figures/Supplementary Figure 2/X.camtschadalis.jpg]

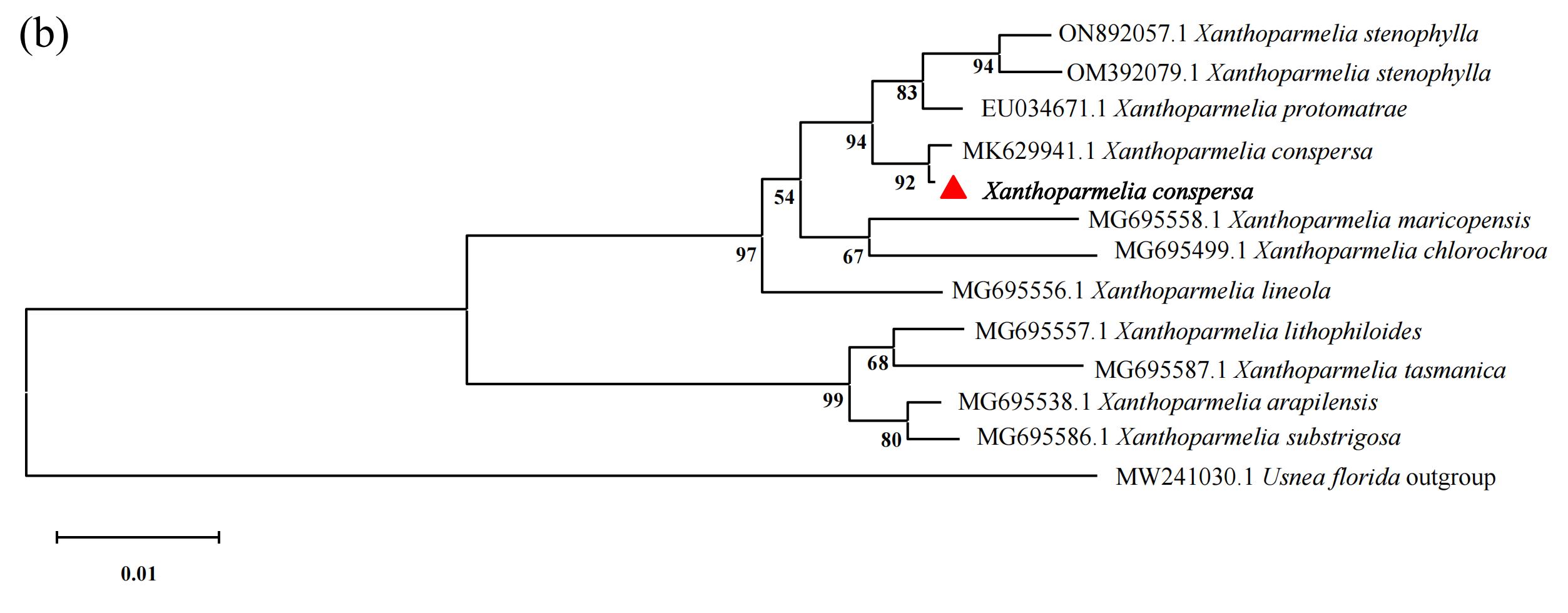

Supplement: SUPPLEMENTARY FIGURE S1 — External morphology of eleven Xanthoparmelia species. [file Data_Sheet_1.zip › Supplementary Figures/Supplementary Figure 2/X.conspersa.jpg]

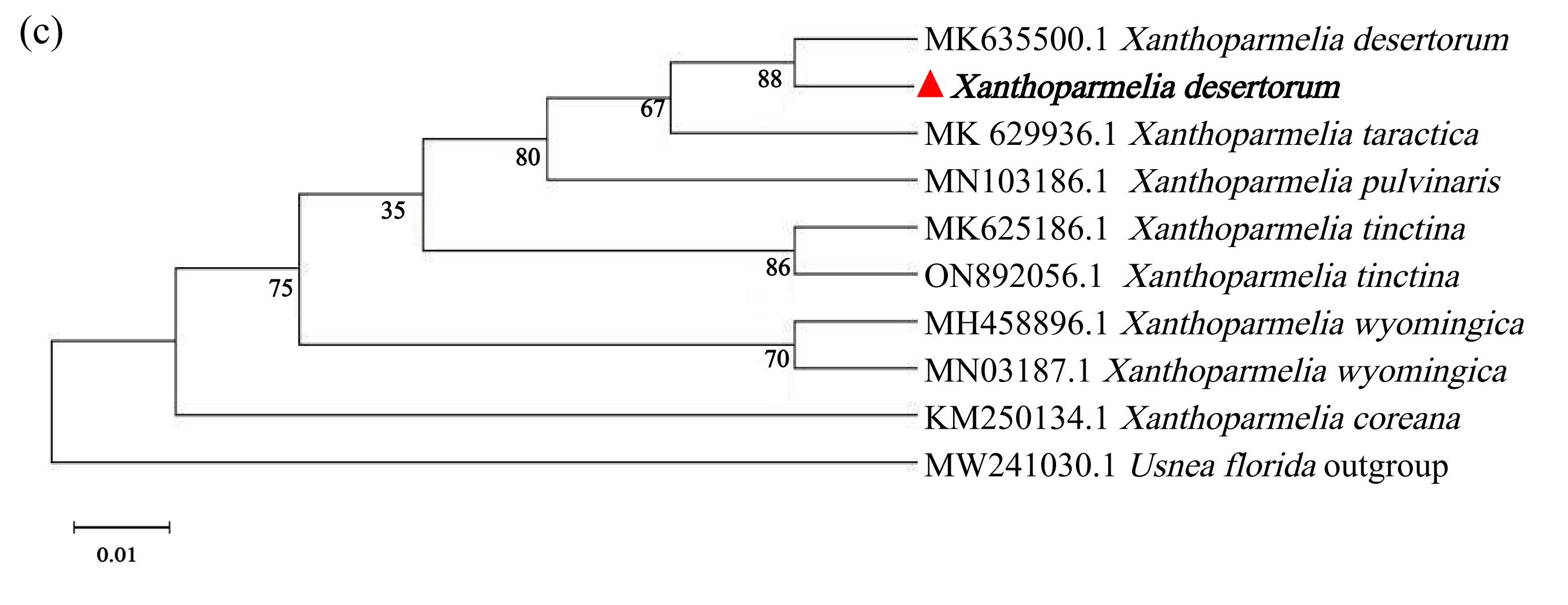

Supplement: SUPPLEMENTARY FIGURE S1 — External morphology of eleven Xanthoparmelia species. [file Data_Sheet_1.zip › Supplementary Figures/Supplementary Figure 2/X.desertorum.jpg]

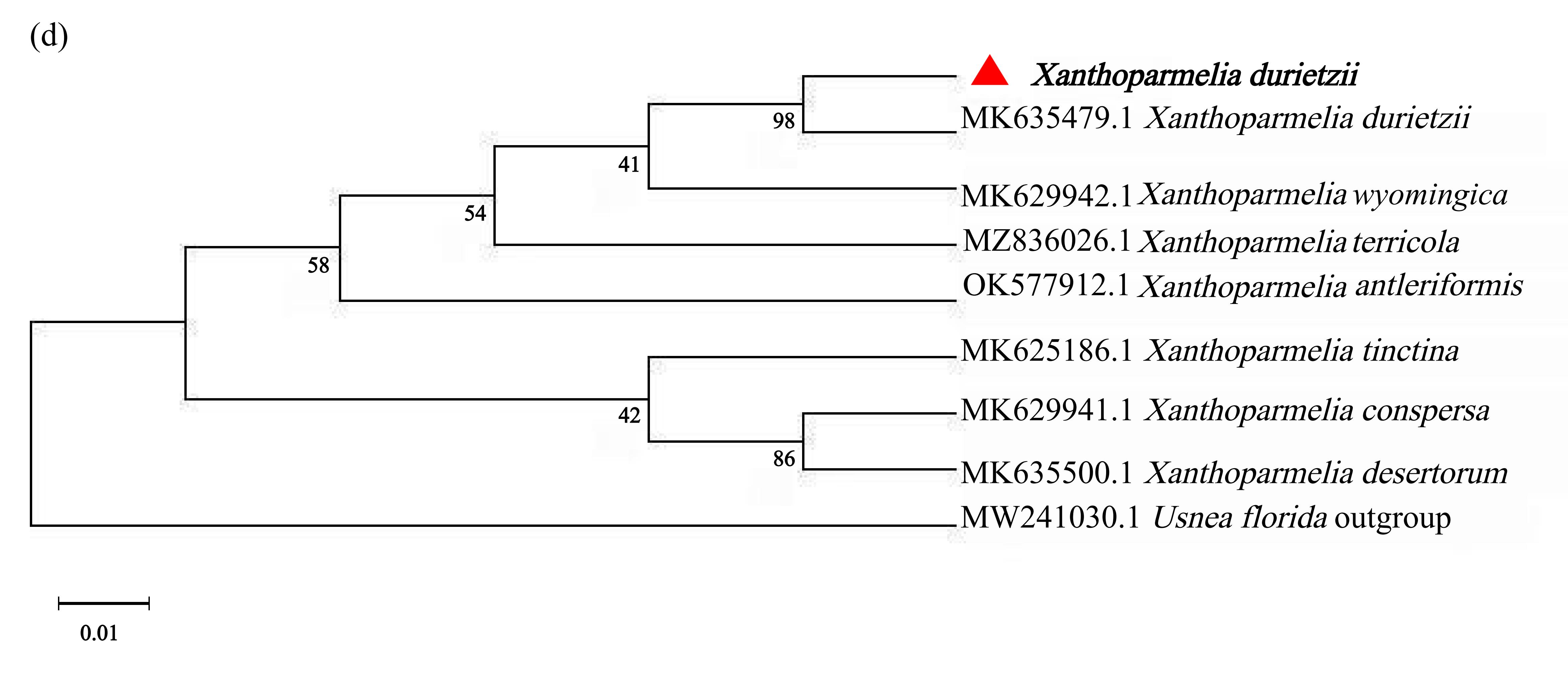

Supplement: SUPPLEMENTARY FIGURE S1 — External morphology of eleven Xanthoparmelia species. [file Data_Sheet_1.zip › Supplementary Figures/Supplementary Figure 2/X.durietzii.jpg]

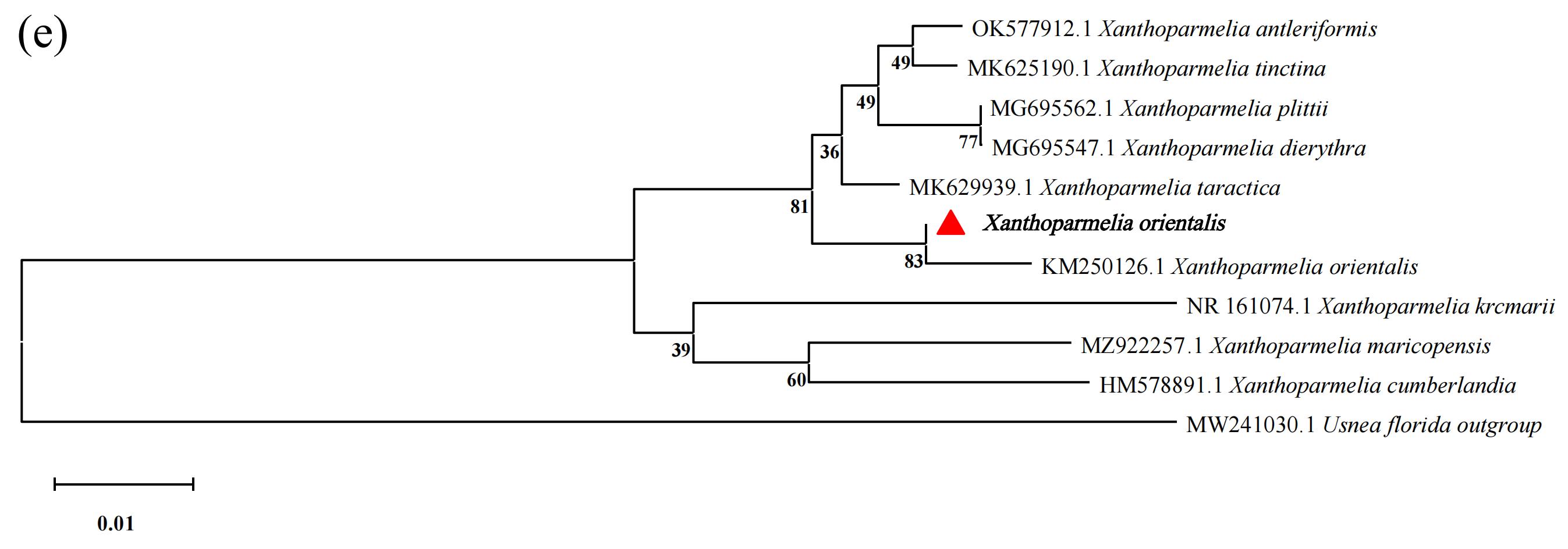

Supplement: SUPPLEMENTARY FIGURE S1 — External morphology of eleven Xanthoparmelia species. [file Data_Sheet_1.zip › Supplementary Figures/Supplementary Figure 2/X.orientalis.jpg]

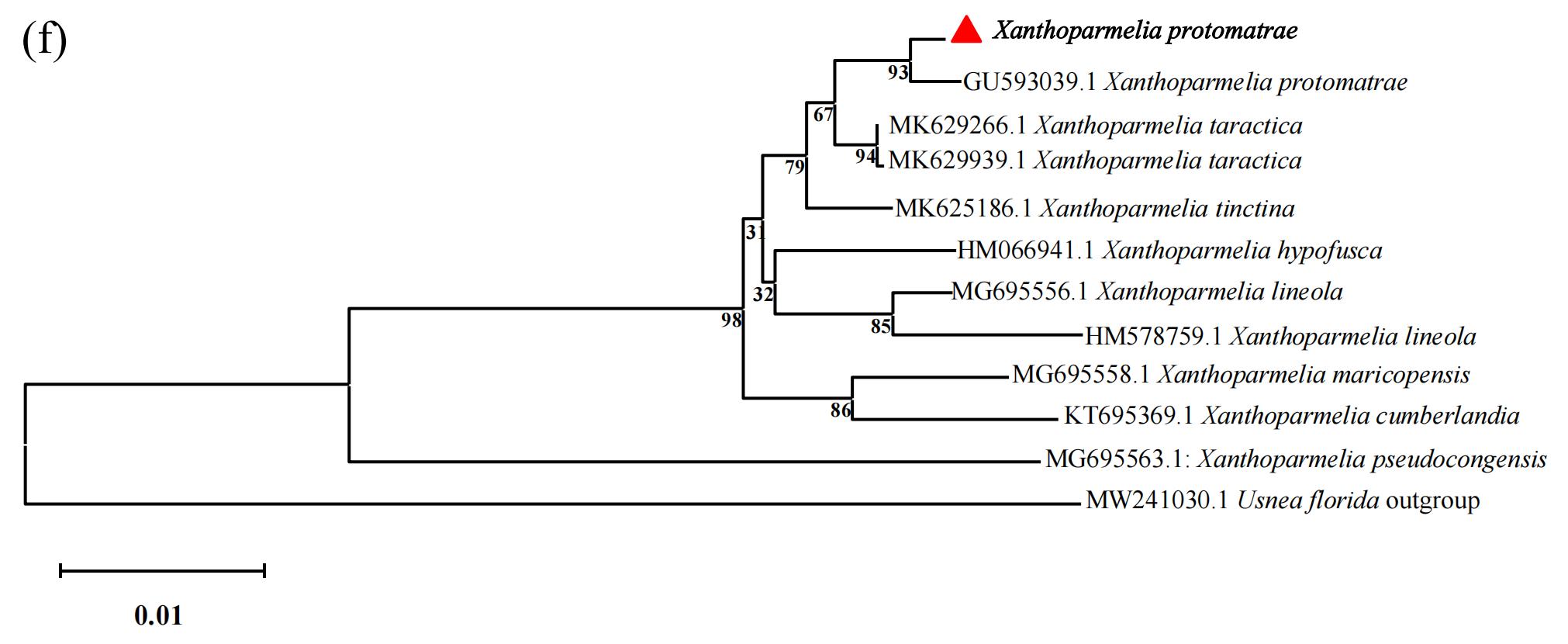

Supplement: SUPPLEMENTARY FIGURE S1 — External morphology of eleven Xanthoparmelia species. [file Data_Sheet_1.zip › Supplementary Figures/Supplementary Figure 2/X.protomatrae.jpg]

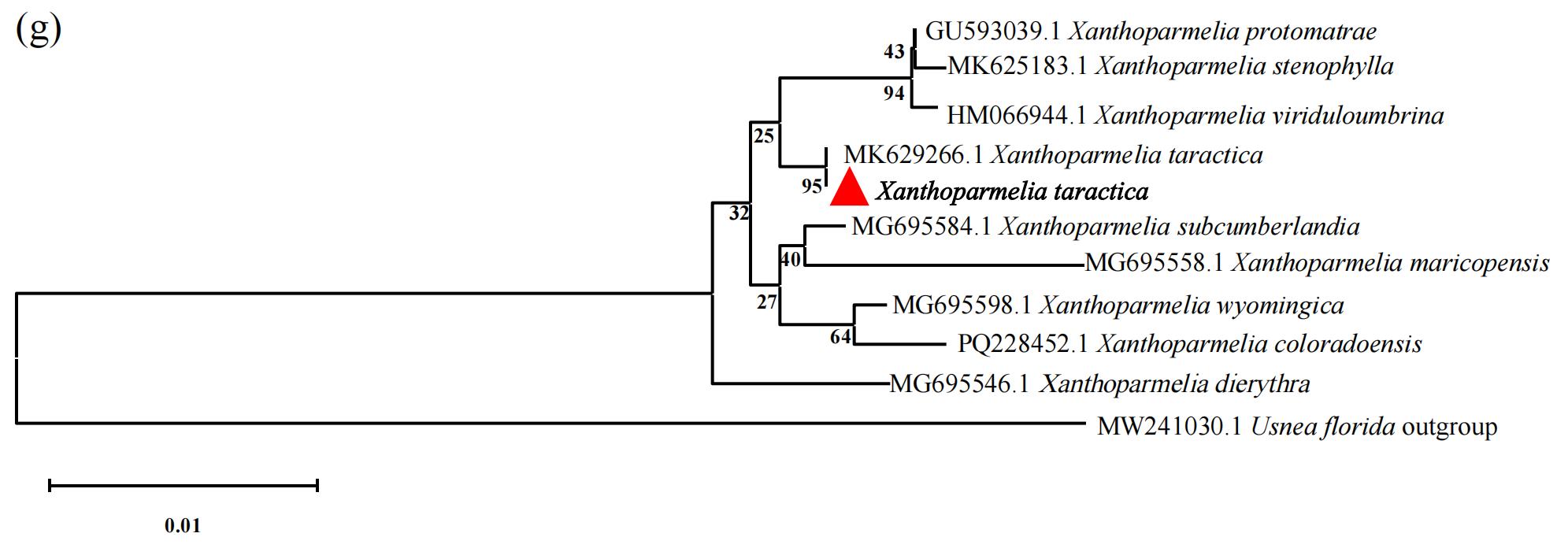

Supplement: SUPPLEMENTARY FIGURE S1 — External morphology of eleven Xanthoparmelia species. [file Data_Sheet_1.zip › Supplementary Figures/Supplementary Figure 2/X.taractica.jpg]

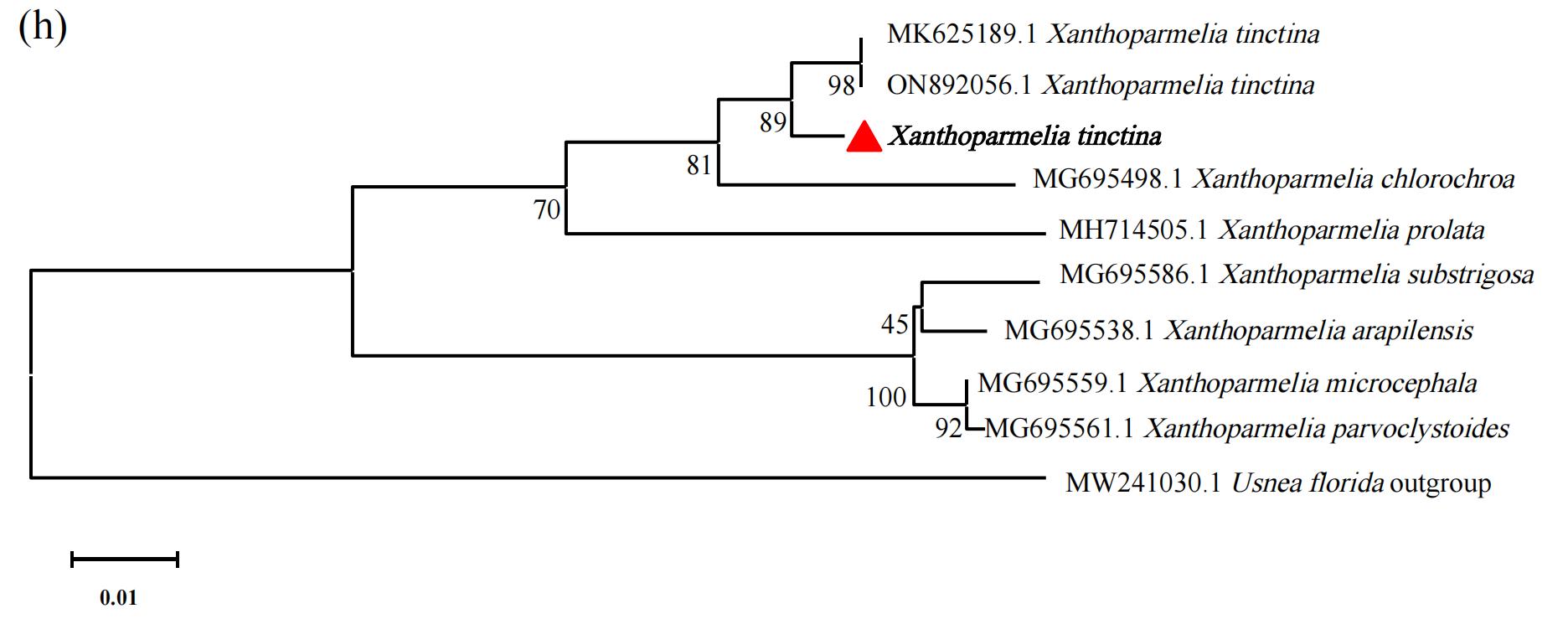

Supplement: SUPPLEMENTARY FIGURE S1 — External morphology of eleven Xanthoparmelia species. [file Data_Sheet_1.zip › Supplementary Figures/Supplementary Figure 2/X.tinctina.jpg]

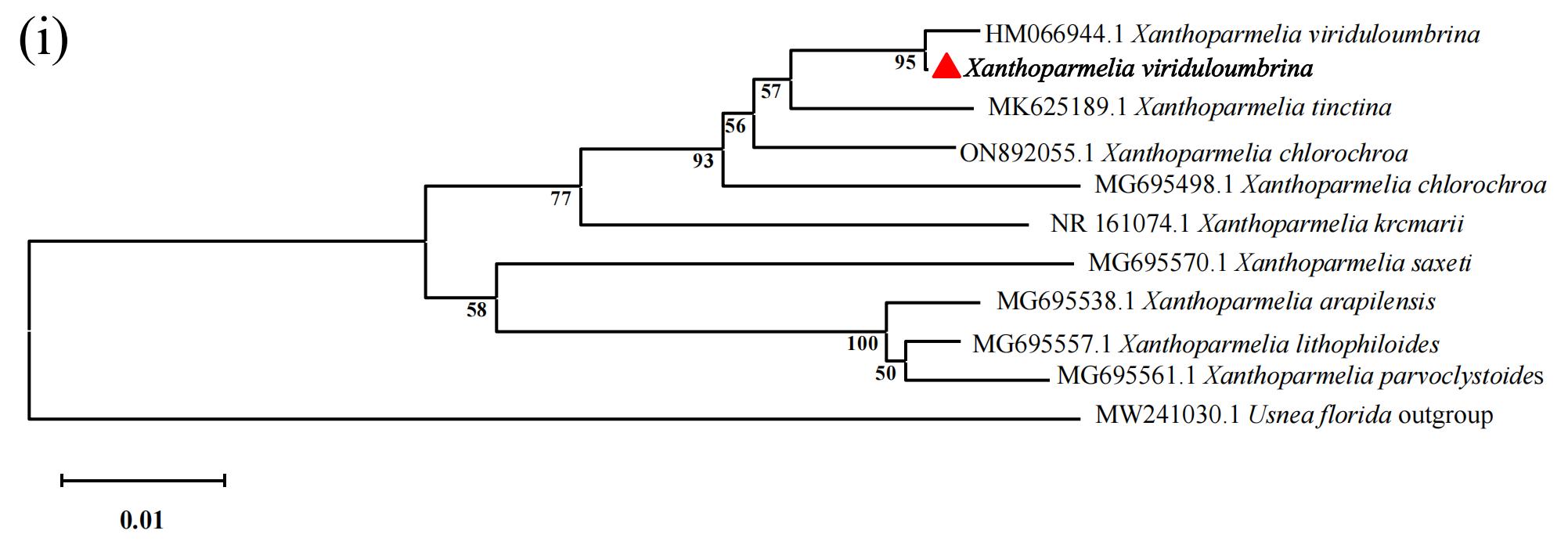

Supplement: SUPPLEMENTARY FIGURE S1 — External morphology of eleven Xanthoparmelia species. [file Data_Sheet_1.zip › Supplementary Figures/Supplementary Figure 2/X.viriduloumbrina.jpg]

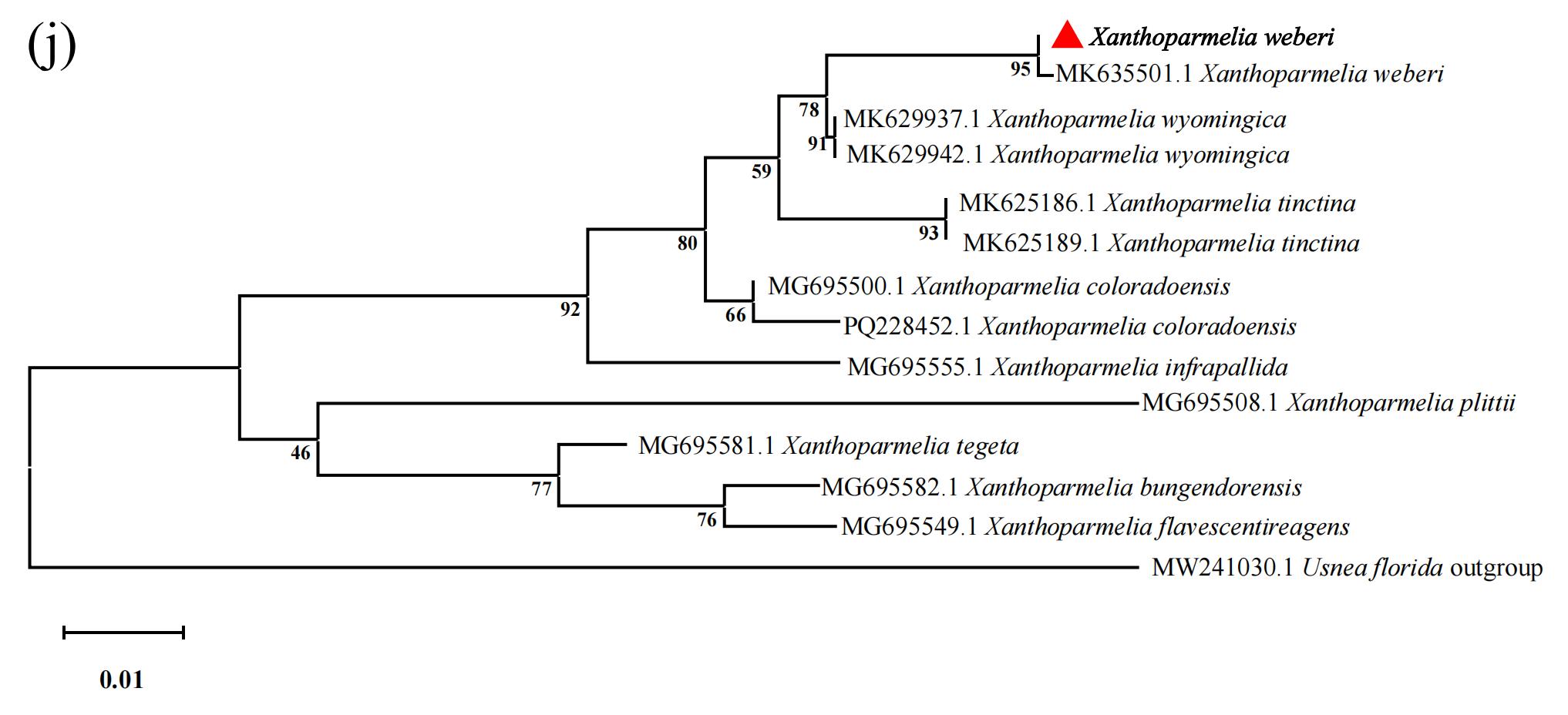

Supplement: SUPPLEMENTARY FIGURE S1 — External morphology of eleven Xanthoparmelia species. [file Data_Sheet_1.zip › Supplementary Figures/Supplementary Figure 2/X.weberi.jpg]

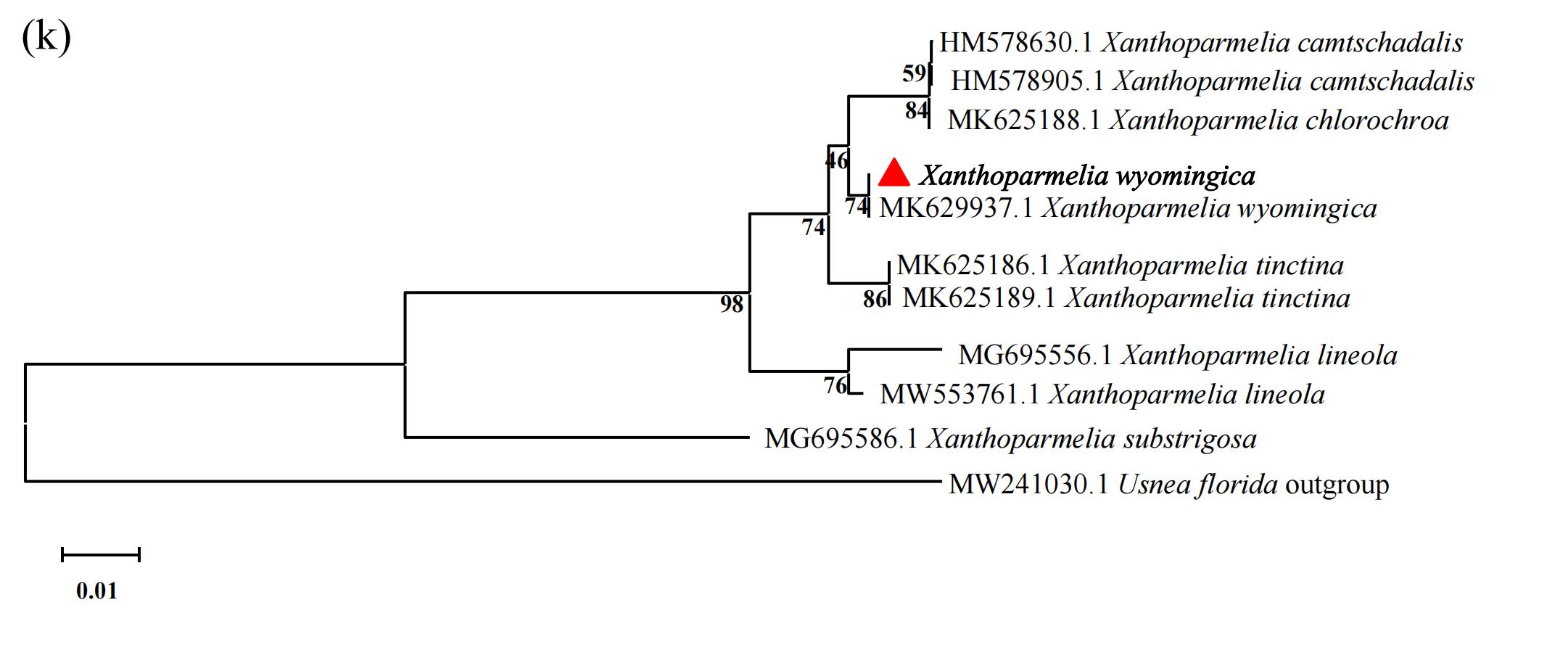

Supplement: SUPPLEMENTARY FIGURE S1 — External morphology of eleven Xanthoparmelia species. [file Data_Sheet_1.zip › Supplementary Figures/Supplementary Figure 2/X.wyomingica.jpg]

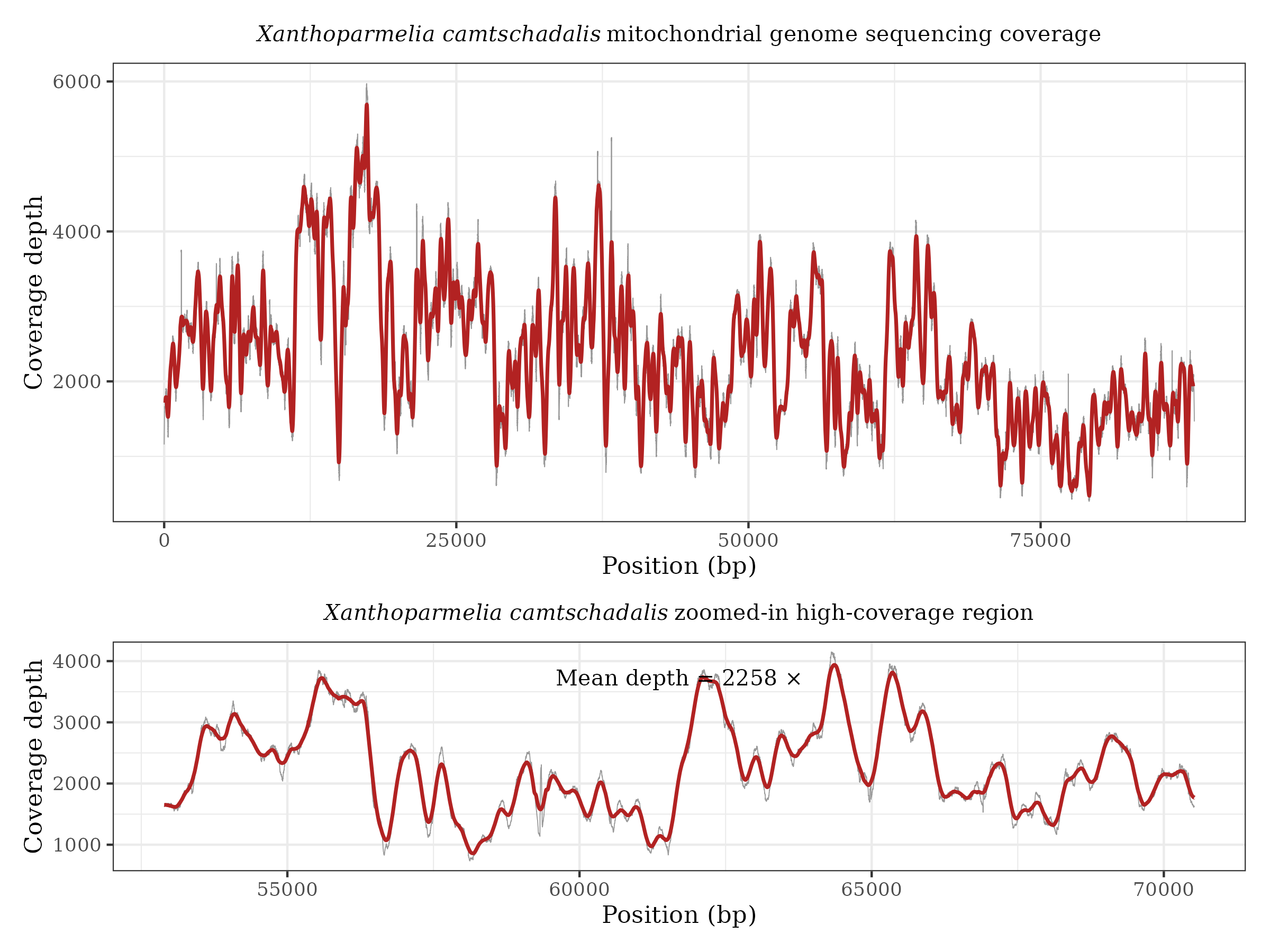

Supplement: SUPPLEMENTARY FIGURE S1 — External morphology of eleven Xanthoparmelia species. [file Data_Sheet_1.zip › Supplementary Figures/Supplementary Figure 3/X.camtschadalis .png]

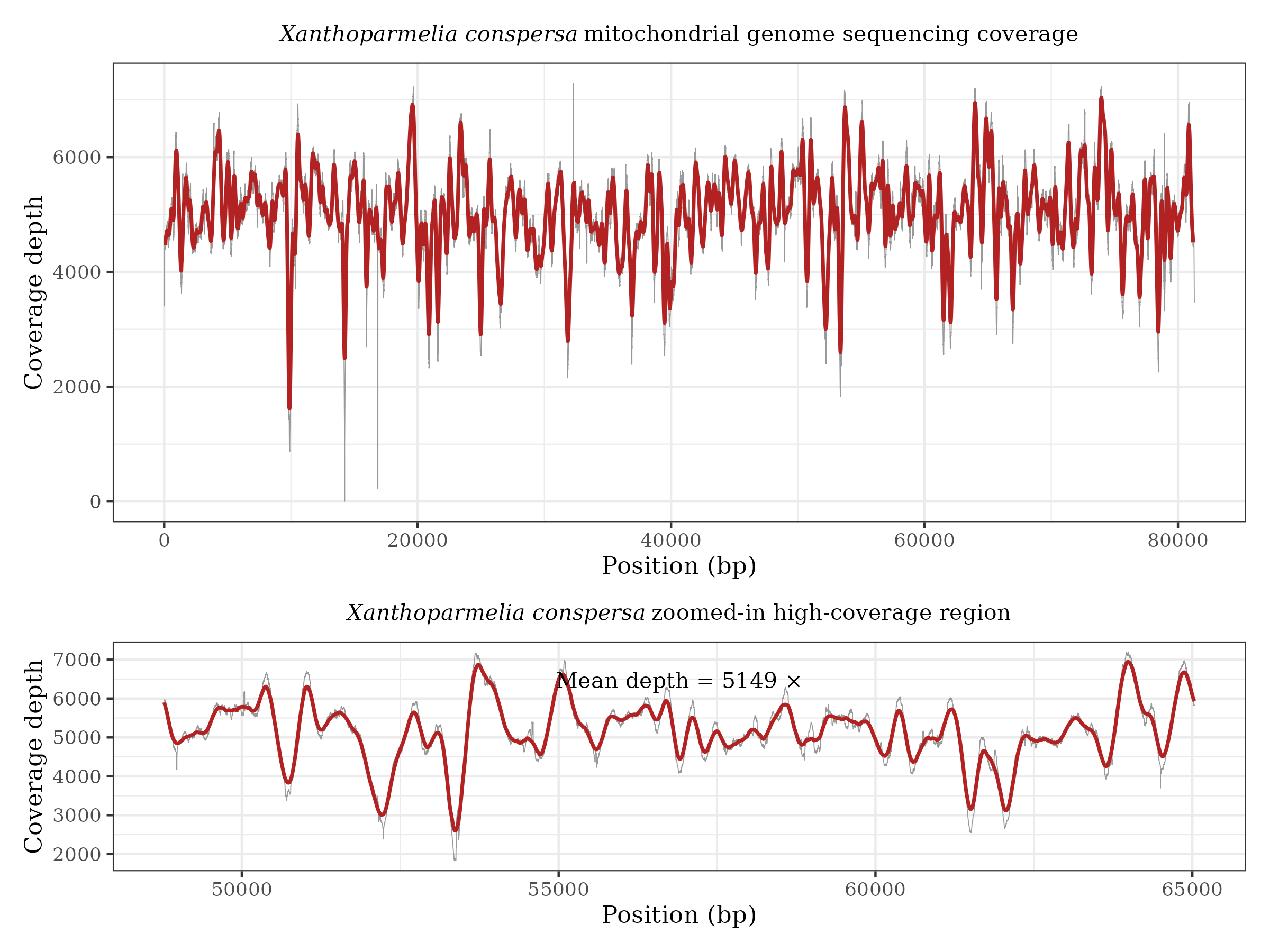

Supplement: SUPPLEMENTARY FIGURE S1 — External morphology of eleven Xanthoparmelia species. [file Data_Sheet_1.zip › Supplementary Figures/Supplementary Figure 3/X.conspersa .png]

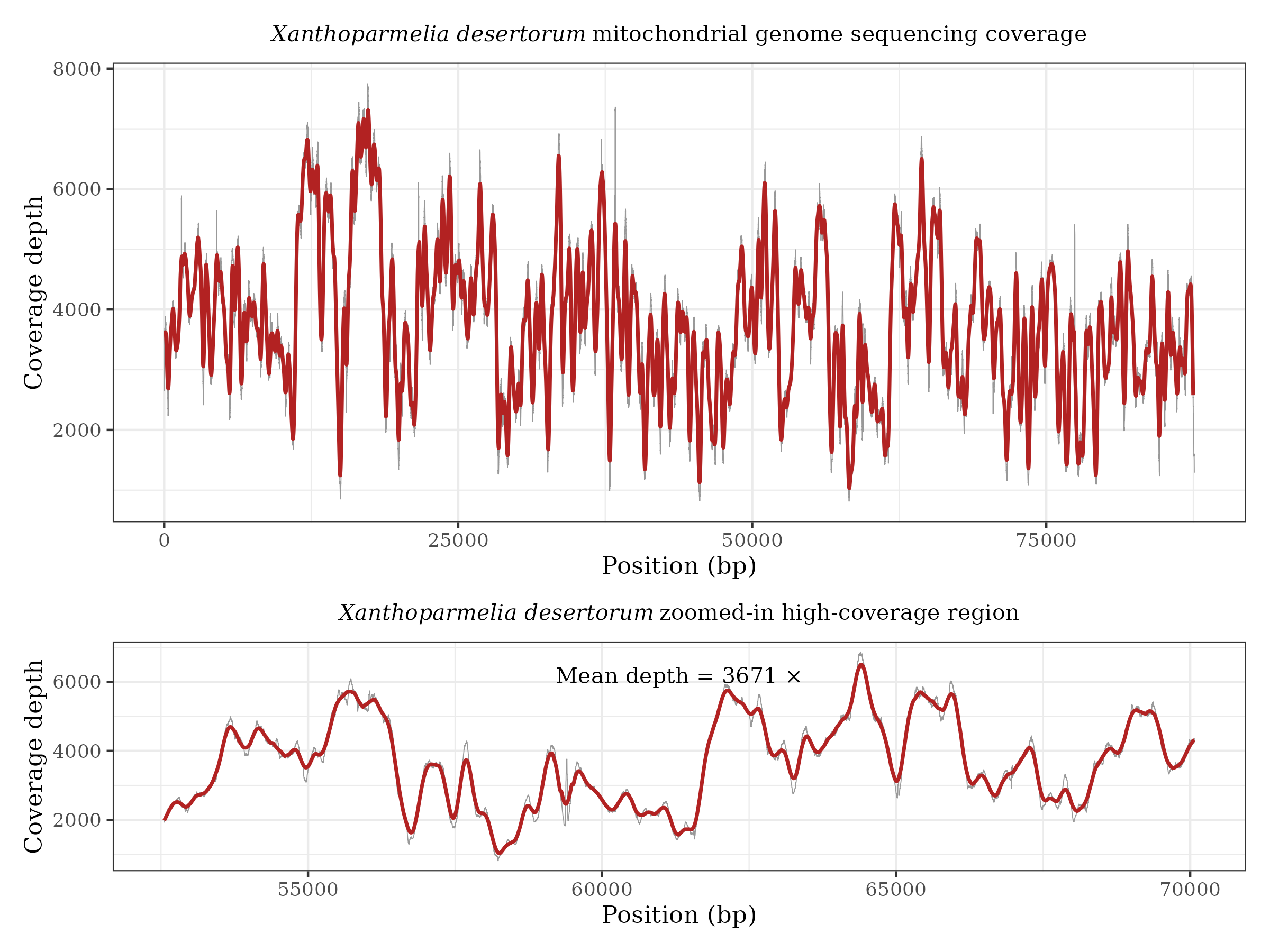

Supplement: SUPPLEMENTARY FIGURE S1 — External morphology of eleven Xanthoparmelia species. [file Data_Sheet_1.zip › Supplementary Figures/Supplementary Figure 3/X.desertorum .png]

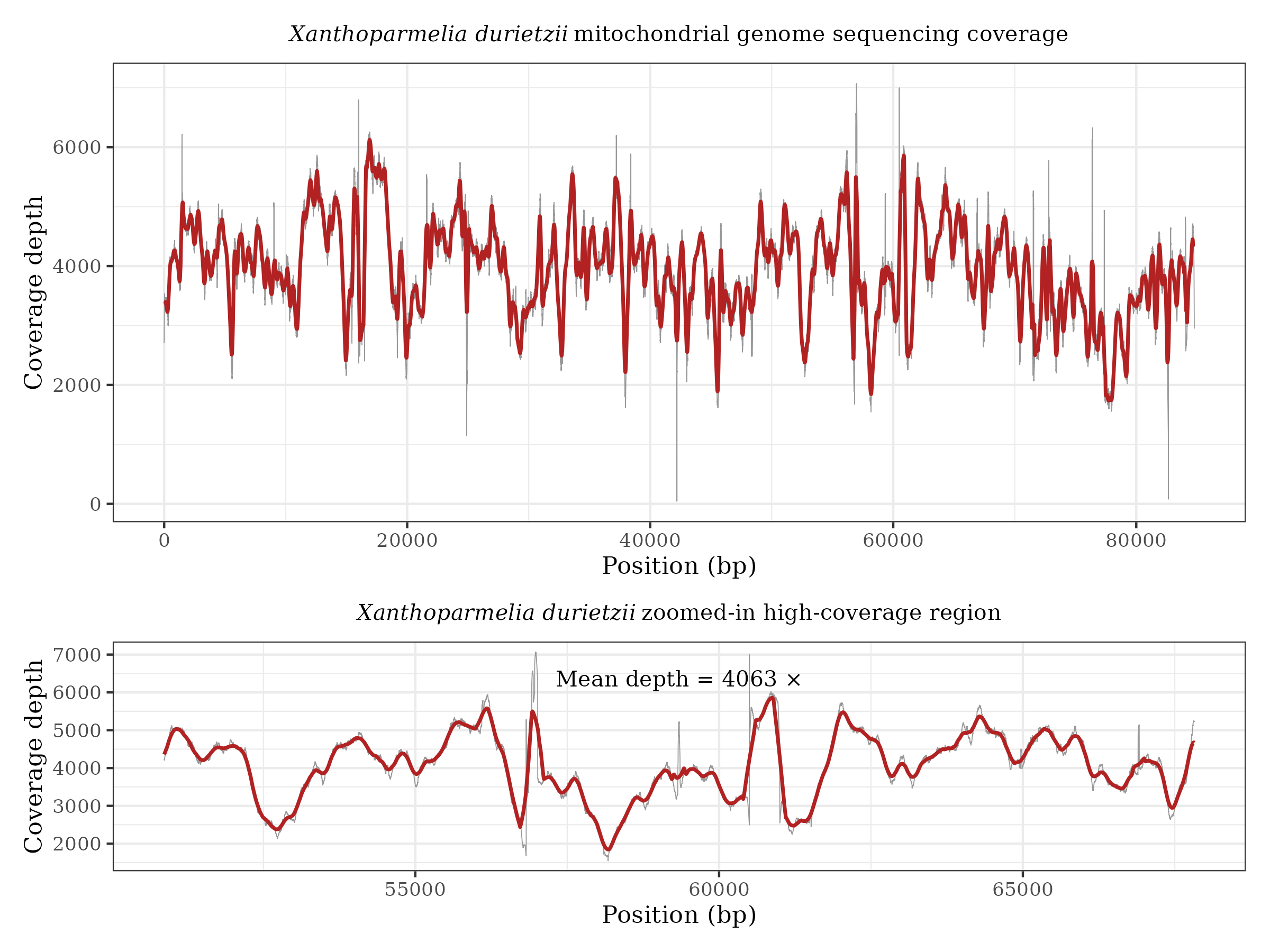

Supplement: SUPPLEMENTARY FIGURE S1 — External morphology of eleven Xanthoparmelia species. [file Data_Sheet_1.zip › Supplementary Figures/Supplementary Figure 3/X.durietzii .png]

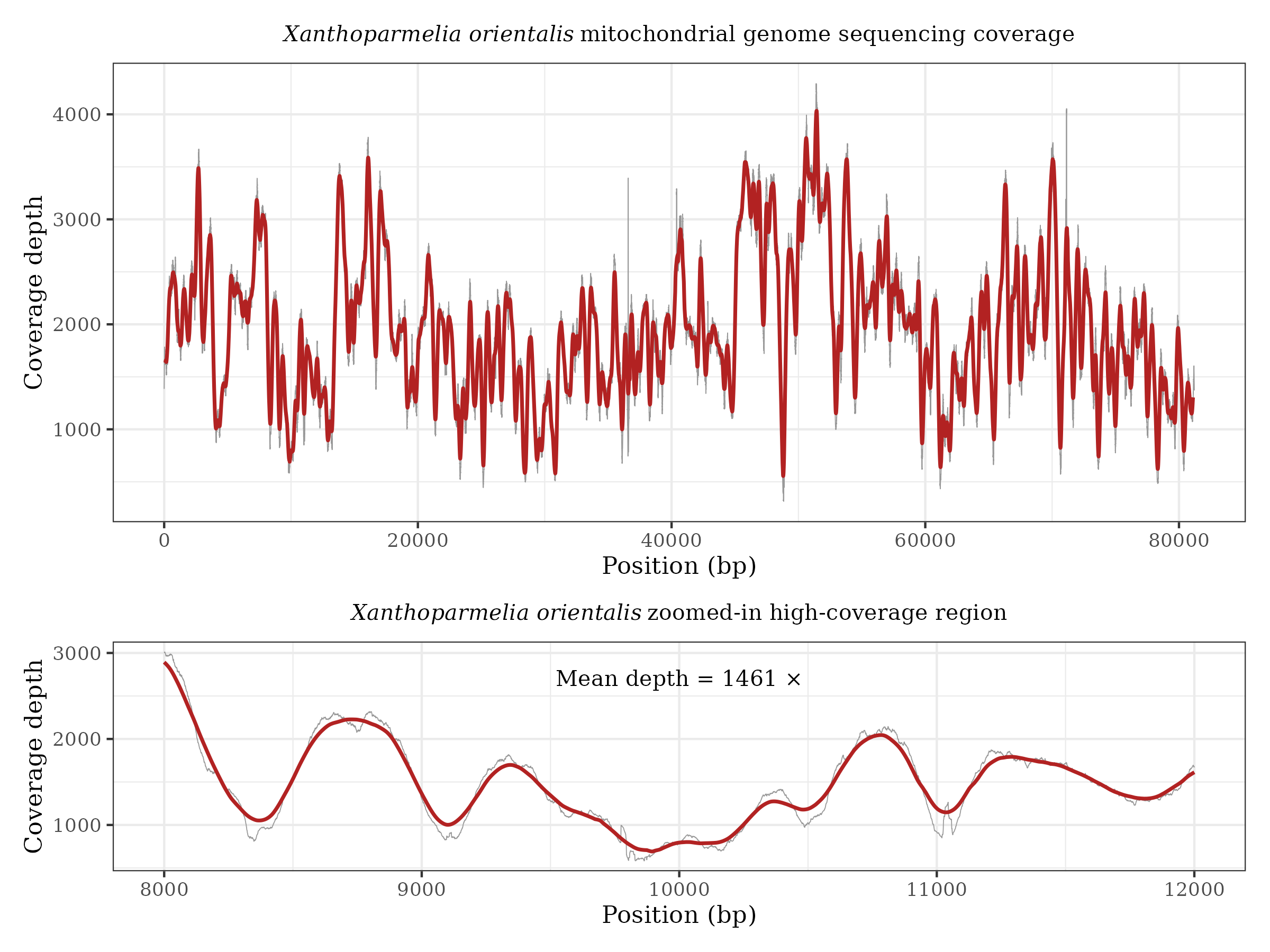

Supplement: SUPPLEMENTARY FIGURE S1 — External morphology of eleven Xanthoparmelia species. [file Data_Sheet_1.zip › Supplementary Figures/Supplementary Figure 3/X.orientalis .png]

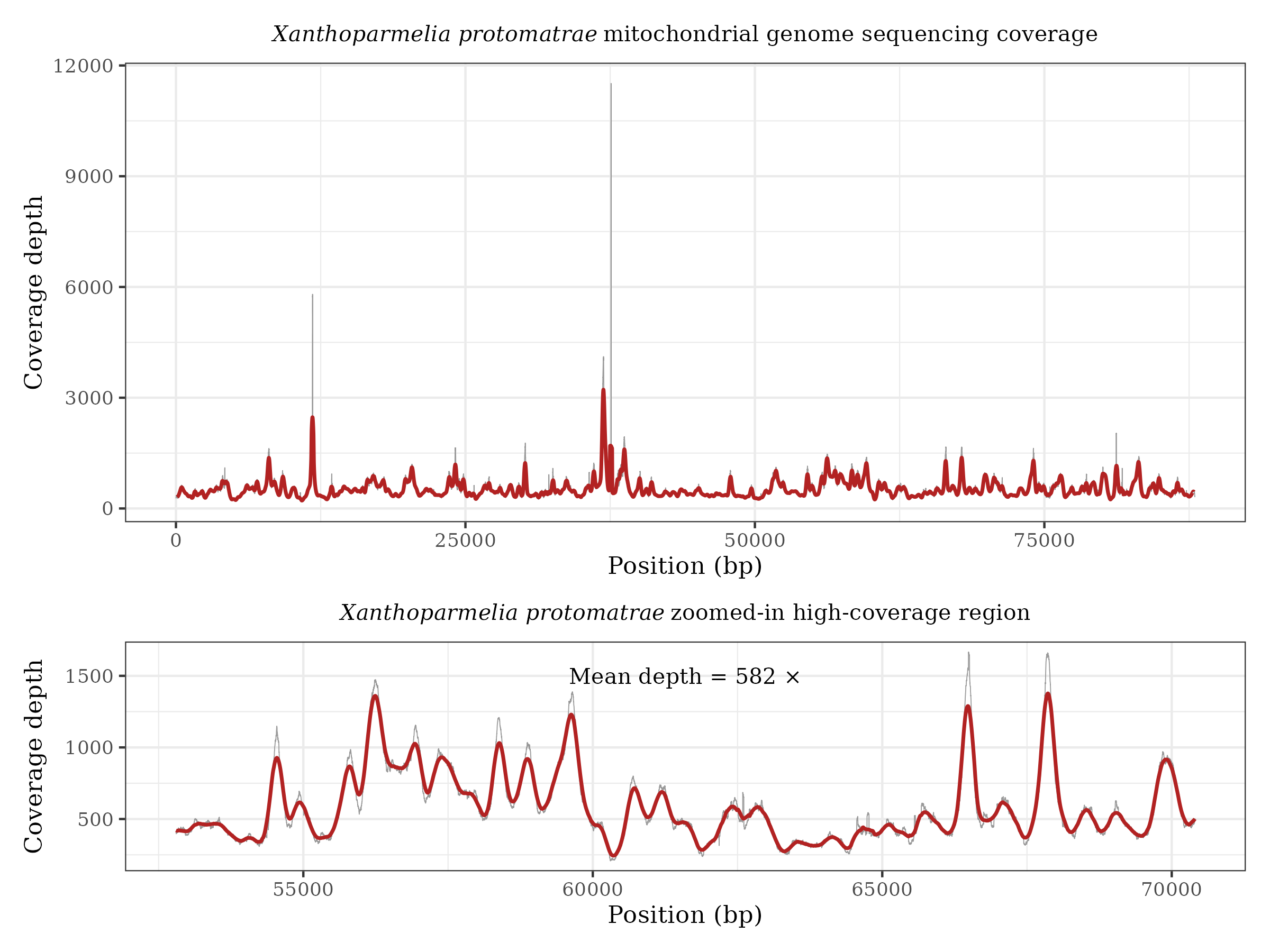

Supplement: SUPPLEMENTARY FIGURE S1 — External morphology of eleven Xanthoparmelia species. [file Data_Sheet_1.zip › Supplementary Figures/Supplementary Figure 3/X.protomatrae .png]

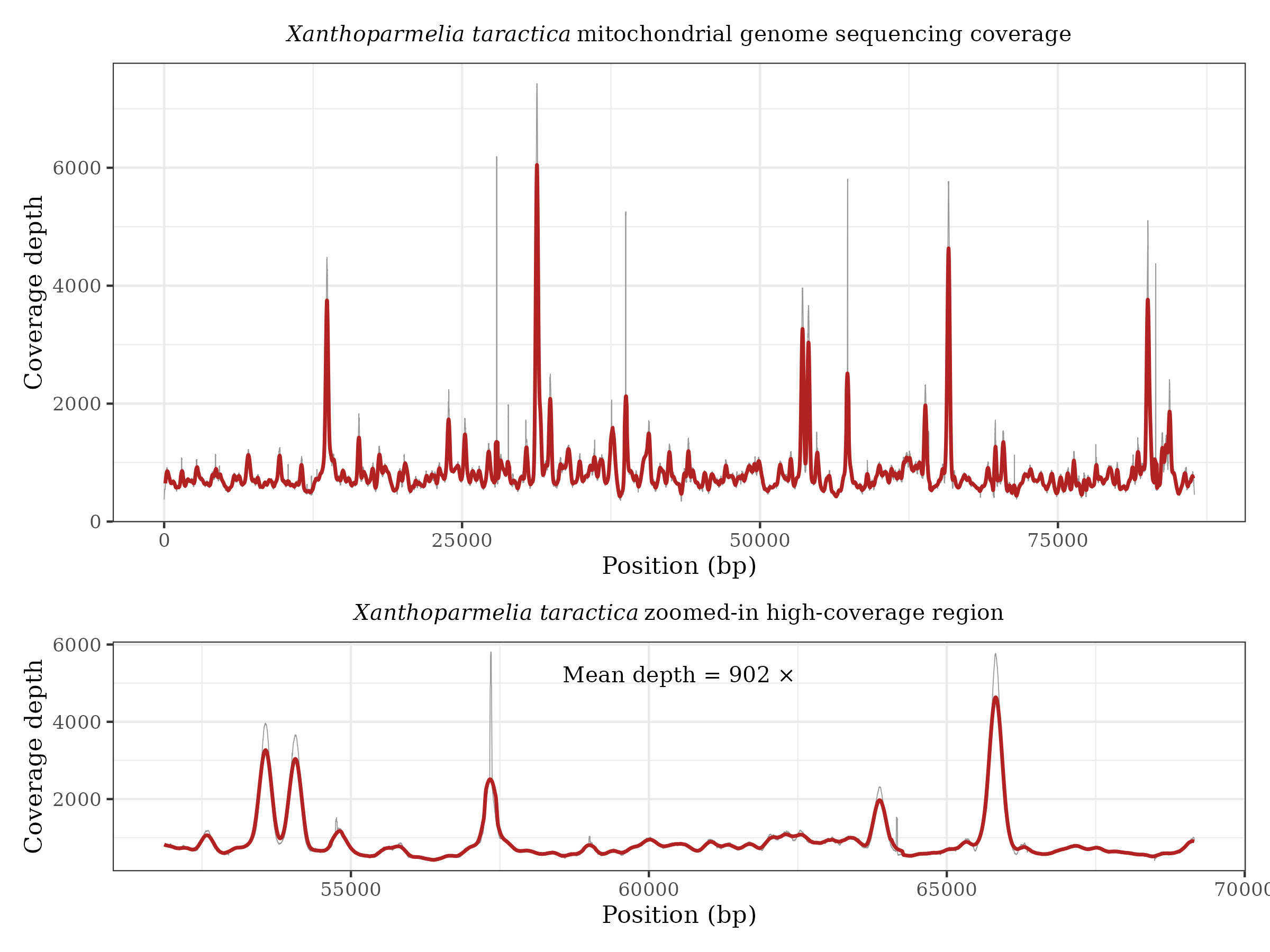

Supplement: SUPPLEMENTARY FIGURE S1 — External morphology of eleven Xanthoparmelia species. [file Data_Sheet_1.zip › Supplementary Figures/Supplementary Figure 3/X.taractica .png]

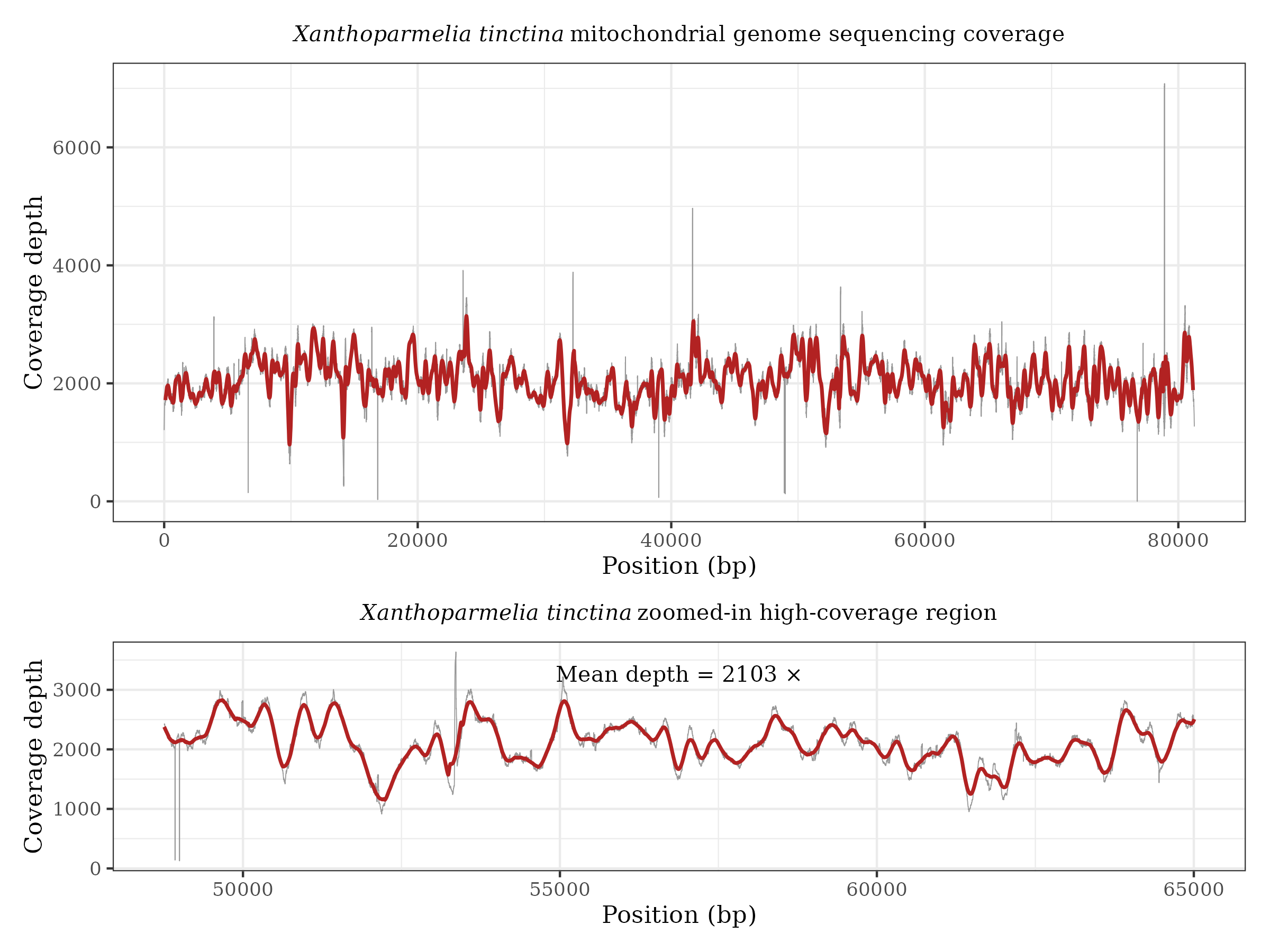

Supplement: SUPPLEMENTARY FIGURE S1 — External morphology of eleven Xanthoparmelia species. [file Data_Sheet_1.zip › Supplementary Figures/Supplementary Figure 3/X.tinctina .png]

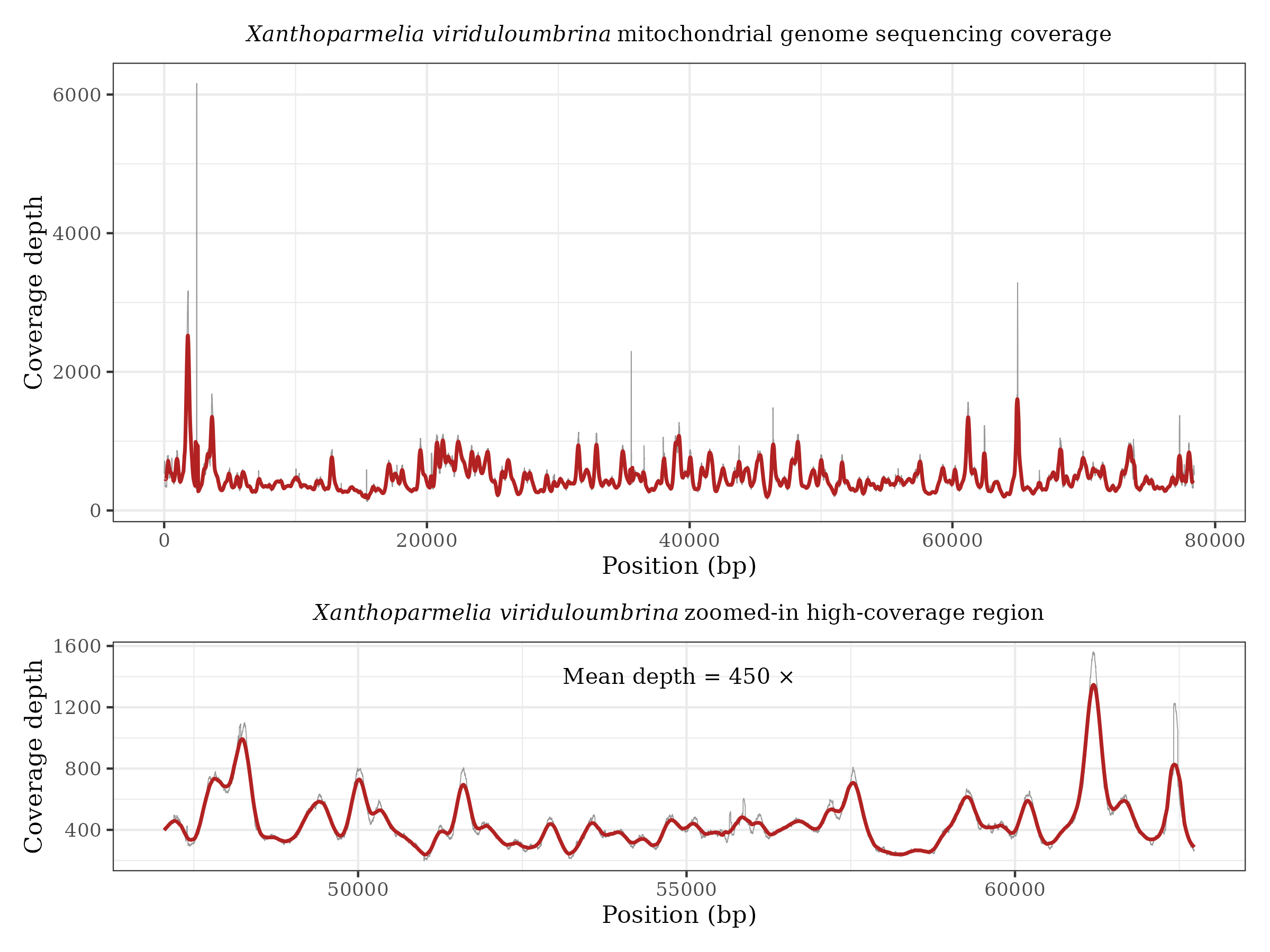

Supplement: SUPPLEMENTARY FIGURE S1 — External morphology of eleven Xanthoparmelia species. [file Data_Sheet_1.zip › Supplementary Figures/Supplementary Figure 3/X.viriduloumbrina .png]

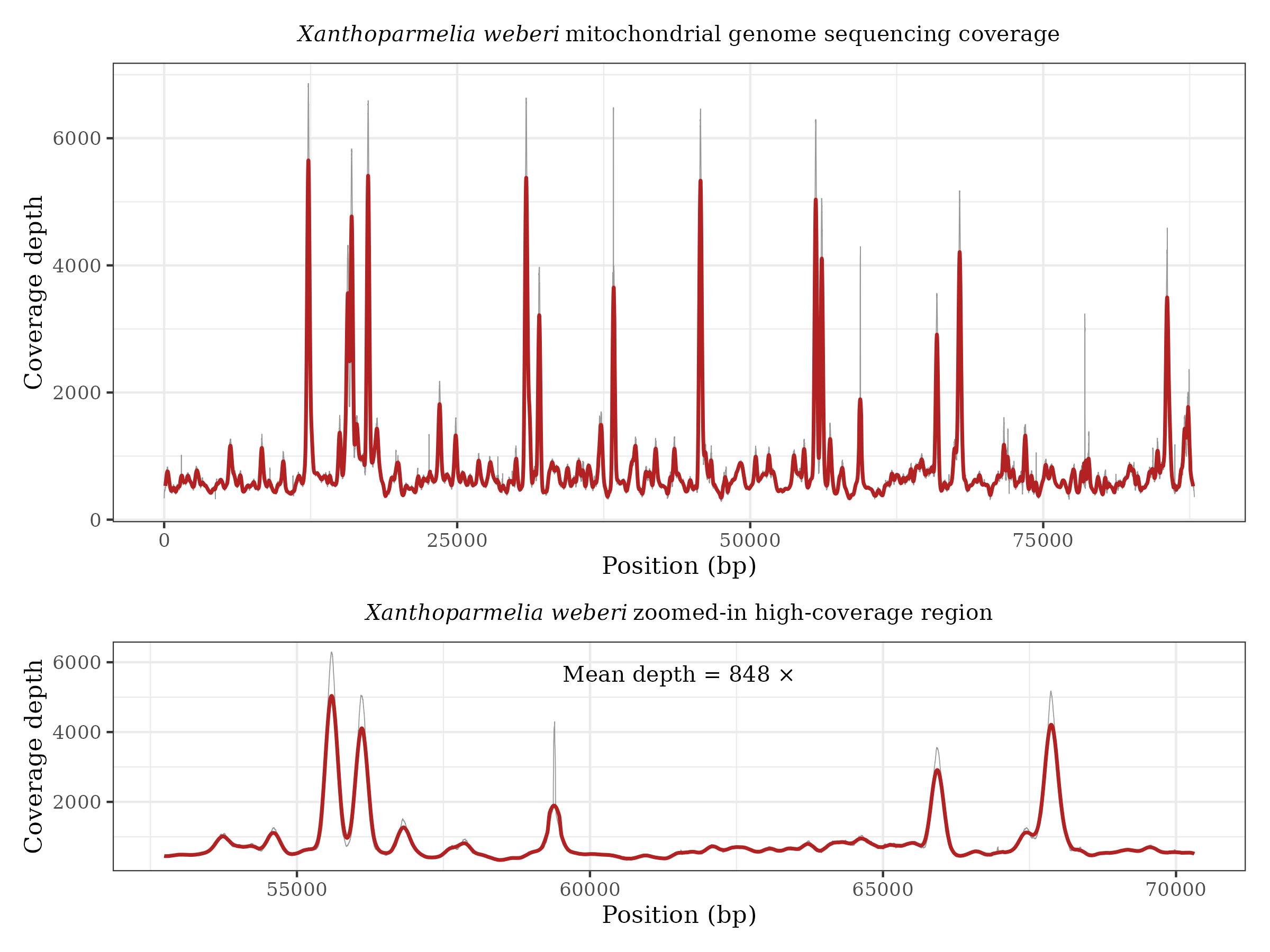

Supplement: SUPPLEMENTARY FIGURE S1 — External morphology of eleven Xanthoparmelia species. [file Data_Sheet_1.zip › Supplementary Figures/Supplementary Figure 3/X.weberi .png]

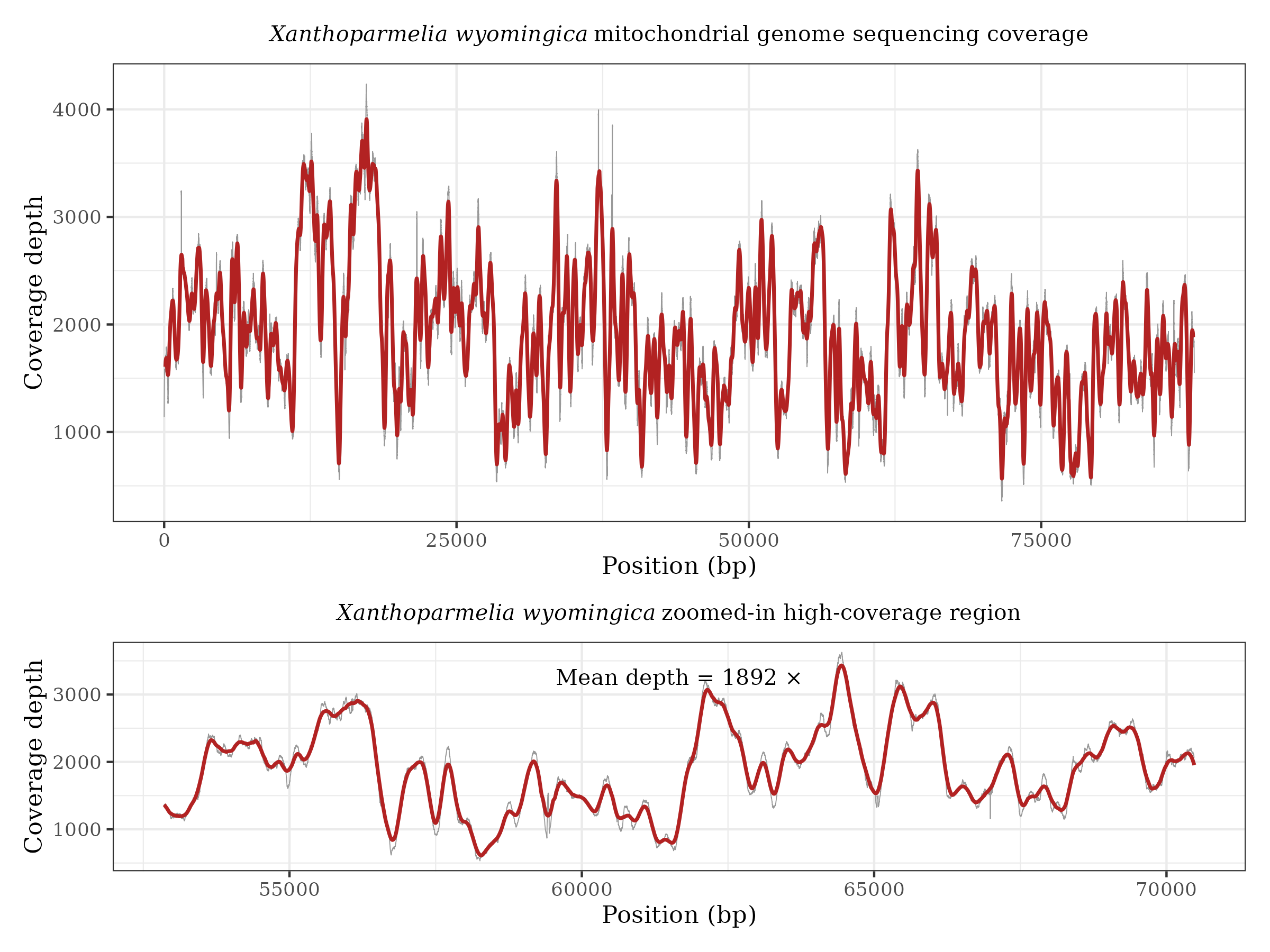

Supplement: SUPPLEMENTARY FIGURE S1 — External morphology of eleven Xanthoparmelia species. [file Data_Sheet_1.zip › Supplementary Figures/Supplementary Figure 3/X.wyomingica .png]
